# A model to simulate human cardio-respiratory responses to different fluid resuscitation treatments after hemorrhagic injury

**DOI:** 10.3389/fphys.2025.1613874

**Published:** 2025-07-01

**Authors:** Varghese Kurian, Xin Jin, Sridevi Nagaraja, Anders Wallqvist, Jaques Reifman

**Affiliations:** ^1^ Department of Defense Biotechnology High Performance Computing Software Applications Institute, Defense Health Agency Research and Development, Medical Research and Development Command, Fort Detrick, MD, United States; ^2^ The Henry M. Jackson Foundation for the Advancement of Military Medicine, Inc., Bethesda, MD, United States

**Keywords:** cardiovascular system, mathematical model, hemorrhage, resuscitation fluid types, oxygen delivery, transcapillary fluid exchange

## Abstract

Decision-support systems based on artificial intelligence and machine learning algorithms can enhance the capability and capacity of medics to provide care for combat casualties during large-scale combat operations. The training and validation of such algorithms require large amounts of vital-sign data, which can be generated using computational models with the appropriate fidelity. Previously, we developed and validated a human cardio-respiratory (CR) model that captures the essential features of the cardiovascular and respiratory responses to hemorrhage and fluid resuscitation. Here, we extended the CR model by adding oxygen transport and fluid exchange between the capillaries and the interstitial space, which allowed us to represent the effect of different resuscitation fluid types, including saline, blood, and blood products, on vital signs and blood variables. We calibrated and validated the model using hemorrhagic-injury and resuscitation data from four experimental swine studies, involving six different types of resuscitation fluids. We captured the general trend of the experimental vital signs and blood variables with average root mean square errors of 6.91 mmHg for mean arterial pressure, 0.49 L/min for cardiac output, 0.72 g/dL for hemoglobin, and 0.70 mL/(kg·min) for delivered oxygen. In addition, model simulations showed that oxygen delivery increased during fluid resuscitation, regardless of the resuscitation fluid type. The extended CR model, with its ability to account for responses to the most widely used resuscitation fluids, will allow us to generate more realistic synthetic data of trauma casualties.

## 1 Introduction

Hemorrhage remains the leading cause of preventable trauma-induced death on the battlefield (Eastridge et al., 2012; Kotwal et al., 2018; Eastridge et al., 2019). Identifying combat casualties at high risk for uncontrolled bleeding, providing timely care, and reducing evacuation time to less than 60 min have considerably increased Warfighter survivability (Dolan et al., 2021). However, in future large-scale combat operations (LSCO) with a near peer in an increasingly contested battlefield with a large number of casualties and delayed evacuation, combat medics will need to provide prolonged casualty care in a resource-constrained environment (Rasmussen et al., 2015; Kharod et al., 2019; Dolan et al., 2021).

Recent advancements in the development of decision-support systems based on artificial intelligence (AI) and machine learning (ML) algorithms can augment the capability and capacity of combat medics to monitor, triage, diagnose, and treat combat casualties near the point of injury (Jin et al., 2017; Jin et al., 2018; Craca et al., 2023; Peng et al., 2023; Stallings et al., 2023; Jin et al., 2024; Keller et al., 2024). However, the training and validation of AI and ML algorithms require massive amounts of curated data, which are not readily available from animal studies due to their small sample size (Sondeen et al., 2011; Simovic et al., 2023) or from clinical studies due to the limited number of measured variables (Liu et al., 2015; Reisner et al., 2016). An alternative solution is to create a synthetic database of battlefield injury and treatment scenarios using validated computational models representing human physiology.

Over the last few decades, several models of the cardio-respiratory system have been proposed to generate insights into the effect of hemorrhage and fluid resuscitation in healthy individuals. These models vary substantially in both scope and size. The majority of the small or medium-sized models (with the number of parameters on the order of 10–100) either completely lack or simplify one or more of the key components of the cardio-respiratory system (e.g., the interstitial fluid compartment), the neuronal control system, or the respiratory system (Grodins, 1959; Mardel et al., 1995; Drobin and Hahn, 1999; Batzel et al., 2004; Tatara et al., 2007; Beard et al., 2013; Siam et al., 2014; Bighamian et al., 2017). In contrast, some of the larger models describe multiple organ systems and their interactions using thousands of parameters and are too complex to calibrate and integrate into AI and ML algorithms (Hester et al., 2011; Bray et al., 2019). Importantly, none of these models had their vital-sign predictions validated against experimental or clinical data involving both hemorrhage and fluid resuscitation using multiple fluid types.

Recently, our U.S. Department of Defense team developed and validated a human physiological model (the cardio-respiratory CR model) that captures the essential features of the cardiovascular and respiratory responses to hemorrhage, fluid resuscitation, and respiratory perturbations (Jin et al., 2023). Validation studies demonstrated that the CR model yields equal or higher accuracy in predicting vital-sign changes resulting from hemorrhagic injuries and resuscitation treatments compared with other similar-size models as well as larger models of the cardiovascular and respiratory systems (Jin et al., 2023). In addition, the parsimonious nature of the CR model (approximately 100 parameters) allows it to be readily integrated into other applications, such as the training and validation of ML algorithms for optimizing the utilization of resuscitation fluids (Jin et al., 2024).

**FIGURE 1:**
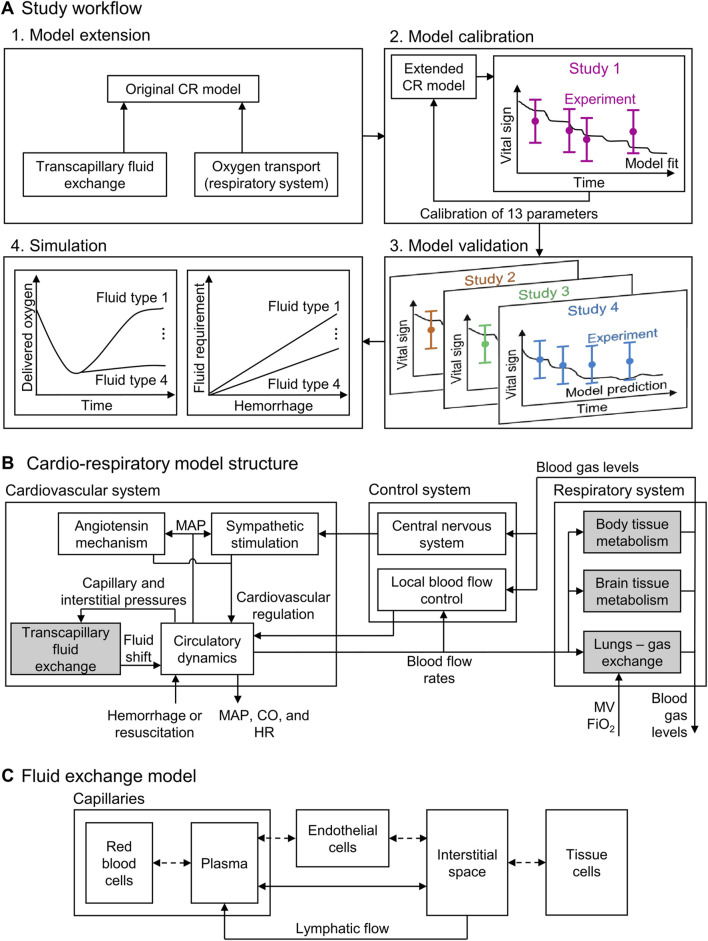
Schematic showing **(A)** the study workflow, including the model extension, model calibration, model validation, and simulation; **(B)** the structure of the cardio-respiratory (CR) model, with the shaded boxes indicating the CR model extensions; and **(C)** the compartments in the fluid exchange model. CO: cardiac output; FiO_2_: fraction of inspired O_2_; HR: heart rate; MAP: mean arterial pressure; MV: minute ventilation.

In the original CR model, we only considered the hydrostatic pressure exerted by a generic resuscitation fluid to predict changes in vital signs resulting from resuscitation interventions after a hemorrhagic injury. This simplification limited the model’s ability to capture the effects of different fluid resuscitation treatments with different salt and protein concentrations. To overcome this limitation, we extended the CR model to represent resuscitation fluids as a mixture of its constituents, including salt and proteins, which allowed us to account for both the hydrostatic and oncotic pressures of saline, blood, and blood products. In addition, we also modified the descriptions of transcapillary fluid exchange and physiology-based oxygen transport in the model (Figure 1A). We calibrated and validated the extended CR model using swine data from four different experimental studies, involving six different types of resuscitation fluids and their effects on vital signs, including mean arterial pressure (MAP), heart rate (HR), and cardiac output (CO), as well as on blood variables, including hemoglobin (Hb) concentration, delivered oxygen (DO_2_), and mixed venous blood oxygen saturation (SvO_2_). Finally, we illustrated the applicability of the model by performing simulations to identify the volume required by these various resuscitation fluid types to reach a given treatment target.

## 2 Methods

### 2.1 Computational model

The original CR model (Jin et al., 2023) represents the cardiovascular and respiratory systems and their regulatory mechanisms (via 74 ordinary differential and algebraic equations with 98 parameters) and predicts the time-dependent evolution of vital signs resulting from hemorrhagic injury and subsequent fluid resuscitation (Figure 1B). Here, we extended the model by incorporating the effects that six different fluid resuscitation types [normal saline (NS), hypertonic saline (HTS), lactated Ringer’s solution (LR), fresh frozen plasma (FFP), fresh whole blood (FWB), and packed red blood cells (PRBC)] transfused following hemorrhage have on the vital signs. To this end, we updated two key elements of the CR model: the transcapillary fluid exchange component and the phenomenological oxygen-dissociation relationship used in modeling the gas exchange and metabolism (Figure 1B, shaded blocks). For a comprehensive overview of the original CR model formulation and implementation, we direct the reader to Jin et al. (2023).

#### 2.1.1 Transcapillary exchange model

The transport of water and solutes between the capillaries and the interstitial space (i.e., the transcapillary fluid exchange) is driven by the differences in both the hydrostatic pressure (previously considered in the CR model) and the oncotic pressure between the two spaces (Kedem and Katchalsky, 1958). To incorporate the effect of oncotic pressure on the transcapillary fluid exchange, we first represented blood as a uniform mixture of hematocrit, plasma proteins, and salt, with the latter two contributing to the oncotic pressure. Next, based on the computational model developed by Mazzoni et al. (1988), we partitioned the capillaries and the space surrounding them into five uniformly mixed compartments (Figure 1C). We divided the space within the capillaries into two compartments, the red blood cells (RBC) and the plasma, and the space outside the capillaries into two compartments, the interstitial space (ISS) and the tissue cells. As the fifth compartment, we represented a layer of endothelial cells that formed the boundary between the capillaries and the ISS. We assumed that the hydrostatic pressure values of RBC, plasma, and endothelial cells as well as those of the ISS and tissue cell compartments were always equal. In addition, we assumed that the membranes separating the five compartments were permeable to water and that the membrane between the capillaries and the ISS also allowed for the transport of salt and albumin.

We used the empirical relationships provided by Mazzoni et al. (1988) and Nitta et al. (1981) to compute the oncotic pressure π of the model compartments for a given average concentration C of proteins and salt in each of the five compartments, as shown in Equations 1–3:

$$\pi^{\text{alb}}=2.8\times 10^{‐1}\left(C^{\text{alb}}\right)+1.8\times 10^{‐3}{\left(C^{\text{alb}}\right)}^{2}+1.2\times 10^{‐5}{\left(C^{\text{alb}}\right)}^{3}$$ (1)

$$\begin{array}{ll} \pi^{\text{glob}} & =2.1\times 10^{‐1}\left(C^{\text{alb}}+C^{\text{glob}}\right)+1.6\times 10^{‐3}{\left(C^{\text{alb}}+C^{\text{glob}}\right)}^{2}\\ & \;+0.9\times 10^{‐6}{\left(C^{\text{alb}}+C^{\text{glob}}\right)}^{3}‐\;\pi^{\text{alb}} \end{array}$$ (2)

$$\pi^{\text{salt}}=6.27\times 10^{2}\left(C^{\text{salt}}\right)$$ (3)

where the superscripts alb and glob denote albumin and globulin, respectively.

We used a modified version of Starling’s equations (Kedem and Katchalsky, 1958) to describe the volumetric flow rate of water J^w^ and the mass flow rates of solutes (J^salt^ for salt and J^alb^ for albumin) across the membranes and used the equations provided by Mazzoni et al. (1988) to describe the lymphatic flow $J_{L}^{w}$ from the ISS to the plasma, as shown in Equations 4–11:

$$J_{\text{Pl}‐\text{EC}}^{w}=L_{\text{Pl}‐\text{EC}}S_{\text{Pl}‐\text{EC}}\left[\pi_{\text{EC}}^{\text{salt}}\;‐\;\left(\pi_{\text{Pl}}^{\text{alb}}+\pi_{\text{Pl}}^{\text{glob}}+\pi_{\text{Pl}}^{\text{salt}}\right)\right]$$ (4)

$$J_{\text{EC}‐\text{ISS}}^{w}=L_{\text{EC}‐\text{ISS}}S_{\text{EC}‐\text{ISS}}\left[P_{\text{Pl}}\;{‐\;P}_{\text{ISS}}+\left(\pi_{\text{ISS}}^{\text{alb}}+{\;\pi }_{\text{ISS}}^{\text{salt}}\right)\;‐\;\pi_{\text{EC}}^{\text{salt}}\right]$$ (5)

$$J_{\text{Pl}‐\text{ISS}}^{w}=L_{\text{Pl}‐\text{ISS}}S_{\text{Pl}‐\text{ISS}}\left[P_{\text{Pl}}\;‐\;P_{\text{ISS}}+\sigma_{\text{Pl}‐\text{ISS}}^{\text{alb}}\left(\pi_{\text{ISS}}^{\text{alb}}\;‐{\;\pi }_{\text{Pl}}^{\text{alb}}\right)+\sigma_{\text{Pl}‐\text{ISS}}^{\text{salt}}\left(\pi_{\text{ISS}}^{\text{salt}}\;‐{\;\pi }_{\text{Cap}}^{\text{salt}}\right)\;‐\;\sigma_{\text{Pl}‐\text{ISS}}^{\text{glob}}\pi_{\text{Pl}}^{\text{glob}}\right]$$ (6)

$$J_{\text{Pl}‐\text{RBC}}^{w}=L_{\text{Pl}‐\text{RBC}}S_{\text{Pl}‐\text{RBC}}\left[\pi_{\text{RBC}}^{\text{salt}}\;‐\;\left(\pi_{\text{Pl}}^{\text{alb}}+\pi_{\text{Pl}}^{\text{glob}}+{\;\pi }_{\text{Cap}}^{\text{salt}}\right)\right]$$ (7)

$$J_{\text{ISS}‐\text{Tcell}}^{w}=L_{\text{ISS}‐\text{Tcell}}S_{\text{ISS}‐\text{Tcell}}\left[\pi_{\text{Tcell}}^{\text{salt}}\;‐\;\left(\pi_{\text{ISS}}^{\text{alb}}+\pi_{\text{ISS}}^{\text{salt}}\right)\right]$$ (8)

$$J_{L}^{w}=\left\{\begin{array}{l} J_{L0}^{w},\\ 7.35P_{\text{ISS}}+J_{L0}^{w},\\ J_{L0}^{w}+29.40, \end{array}\right.\begin{array}{l} P_{\text{ISS}}<0\\ 0\leq P_{\text{ISS}}<4\\ P_{\text{ISS}}\geq 4 \end{array}$$ (9)

$$J_{\text{Pl}‐\text{ISS}}^{\text{salt}}={\bar{C}}_{\text{Pl}‐\text{ISS}}^{\text{salt}}\left(1\;‐\;\sigma_{\text{Pl}‐\text{ISS}}^{\text{salt}}\right)J_{\text{Pl}‐\text{ISS}}^{w}+PS_{\text{Pl}‐\text{ISS}}^{\text{salt}}\Delta C_{\text{Pl}‐\text{ISS}}^{\text{salt}}$$ (10)

$$J_{\text{Pl}‐\text{ISS}}^{\text{alb}}={\bar{C}}_{\text{Pl}‐\text{ISS}}^{\text{alb}}\left(1\;‐\;\sigma_{\text{Pl}‐\text{ISS}}^{\text{alb}}\right)J_{\text{Pl}‐\text{ISS}}^{w}+PS_{\text{Pl}‐\text{ISS}}^{\text{alb}}\Delta C_{\text{Pl}‐\text{ISS}}^{\text{alb}}$$ (11)

where P represents the hydrostatic pressure in a compartment, σ denotes the reflection coefficient of salt or a protein, L represents the hydraulic conductivity and S denotes the total surface area of a membrane, $J_{L0}^{w}$ represents the lymphatic flow at nominal conditions, PS represents the product of the permeability and the surface area of a membrane, and $\bar{C}$ and Δ C represent the average concentration and the difference between concentrations of solutes in two adjacent compartments, respectively. The superscripts denote the type of solute (alb, albumin; glob, globulin; or salt). The subscripts for P and π denote the compartment type (EC, endothelial cells; ISS, interstitial space; Pl, plasma; RBC, red blood cells; or Tcell, tissue cells), and for the other quantities, the subscripts denote the membrane separating two adjacent compartments (e.g., $L_{\text{Pl}‐\text{RBC}}$ denotes the hydraulic conductivity of the membrane separating the plasma and the red blood cells). We also assumed that the concentrations of solutes (albumin and salt) in the lymphatic fluid were equal to those in the ISS.

In the ISS, we defined the change in the hydrostatic pressure from its nominal value ΔP_ISS_ as a function of the change in the ISS volume from its nominal value ΔV_ISS_, as shown in Equation 12:

$${\Delta P}_{\text{ISS}}=\left\{\begin{array}{l} \Delta V_{\text{ISS}}/1.7,\\ \Delta V_{\text{ISS}}/1.2,\\ 2\left(\Delta V_{\text{ISS}}\;‐\;4.8\right)+4, \end{array}\right.\begin{array}{l} \Delta V_{\text{ISS}}<0.0\\ 0.0\leq \Delta V_{\text{ISS}}<4.8\\ \Delta V_{\text{ISS}}\geq 4.8 \end{array}$$ (12)

Finally, based on previous computational studies (Simpson et al., 1996; Batzel et al., 2004; Michel et al., 2020), we applied a constraint on the maximum volume of water that can be absorbed from the ISS into the plasma during hemorrhage. Accordingly, we scaled the volumetric flow rate of water J^w^ in Equations 4, 7 based on the volume of water absorbed $V_{\text{refill}}$ , to compute the final flow rate $J^{w^{\prime }}$ , as shown in Equation 13:

$$J_{j}^{w^{\prime }}=\left\{\begin{array}{ll} J_{j}^{w}{\left(1\;‐\;\frac{V_{\text{refill}}}{V_{\text{refill}}^{\max}}\right)}^{2}, & J_{j}^{w}\leq 0\text{ and }V_{\text{refill}}\geq 0\\ J_{j}^{w}, & \text{otherwise} \end{array}\right.$$

$$V_{\text{refill}}=\int -\left(J_{\text{Pl}-\text{ISS}}^{w^{\prime }}+J_{\text{Pl}-\text{EC}}^{w^{\prime }}\right)\text{dt}$$ (13)

where the subscript j = Pl-ISS or Pl-EC represents the flow from the plasma to the ISS or from the plasma to the endothelial cells, respectively, and $V_{\text{refill}}^{\max}$ denotes the maximum value of $V_{\text{refill}}$ .

#### 2.1.2 Oxygen dissociation model

To model the exchange of oxygen and carbon dioxide that occurs in the lungs and in the body tissues, we calculated the blood oxygen concentration $C_{O2}$ and the carbon dioxide concentration $C_{\text{CO}2}$ for a given partial pressure of these gases. In the original CR model, we described the equations representing this phenomenon under the assumption that the concentration of hemoglobin Hb remained constant. Here, we modified these equations to reflect changes in Hb concentration due to resuscitation with different fluid types. In the modified equations, we assumed that the concentration of oxygen in the blood was proportional to the Hb concentration. However, because carbon dioxide is primarily dissolved in the plasma and its concentration does not change substantially with Hb concentration, we did not update the equations describing its dissolution in blood. The modified Equations 14, 15 are as follows:

$$C_{j,O2}=0.0227c_{1}\frac{F_{j,O2}^{\frac{1}{a_{1}}}}{1+F_{j,O2}^{\frac{1}{a_{1}}}}\frac{\text{Hb}}{Hb_{0}}$$ (14)

$$C_{j,\text{CO}2}=0.0227c_{2}\frac{F_{j,\text{CO}2}^{\frac{1}{a_{2}}}}{1+F_{j,\text{CO}2}^{\frac{1}{a_{2}}}}$$ (15)

where the subscript j represents either arterial or venous blood and Hb_0_ represents the hemoglobin concentration under nominal condition. F_O2_ and F_CO2_ are each a function of both the partial pressure of oxygen and carbon dioxide in the blood, and the constants a_1_, a_2_, c_1_, and c_2_ represent parameters given by Spencer et al. (1979) after fitting the relation to experimental data.

This extension added 30 new equations and 20 new parameters to the original CR model for a total of 104 ordinary differential and algebraic equations and 118 parameters. The model inputs include the rate of hemorrhage, resuscitation fluid type, rate of fluid resuscitation, minute ventilation, and fraction of inspired oxygen. The model generates as outputs the arterial blood pressures [systolic, diastolic, and mean], HR, CO, blood volume (the net volume of fluid in the vasculature), Hb concentration, partial pressure of end-tidal carbon dioxide, and oxygen saturation. The complete set of model equations and the associated parameter values are available in the Supplementary Material, Sections 1 and 2, respectively. We performed all simulations using MATLAB 24.2 (MathWorks, Natick, MA, United States) and solved the model’s system of equations using Euler’s method (Griffiths and Higham, 2010) with a time step of 4.17 × 10^−4^ min.

### 2.2 Model calibration and validation

We selected the four animal experimental studies listed in Table 1 to calibrate and validate the model because their protocols involved a range of hemorrhagic injuries (31%–79% of total blood volume) followed by resuscitation treatment with up to six commonly used fluids. In addition, these studies provided sufficient information for us to computationally simulate the experimental protocols and reported the values of vital signs predicted by the CR model. We selected *Study 1* for model calibration because it had information from three fluid types (i.e., NS, HTS, and FFP), which was sufficient to determine the oncotic pressures and transcapillary exchange of all six fluid types. We selected *Studies 2-4* for model validation because they involved additional fluid types (e.g., FWB), their combination (e.g., a mixture of albumin and HTS), and their administration at different rates, which allowed us to more broadly challenge the model. *Study 4* was conducted more than a decade ago but was never reported in the open literature. The study was conducted at the U.S. Army Institute of Surgical Research (USAISR) in Fort Sam Houston, Texas, in a facility certified by the Association for the Assessment and Accreditation of Laboratory Animal Care International. The study was approved by the Institutional Animal Care and Use Committee of the USAISR and was performed in compliance with the Animal Welfare Act and in accordance with the Guide for the Care and Use of Laboratory Animals. The protocol for this study is described in section of the Supplementary Material.

**TABLE 1:** Studies used for calibration and validation of the extended cardio-respiratory model.

Study (source)		No. of groups	Animals/group	Weight^a^ (kg)	Experiment duration (min)	Hemorrhage volume (%)	Fluid type	Infusion volume (%)	Infusion rate (mL/min)	Outputs
1	Soller et al. (2014) ^b^	3	8	39.1 (2.4)	80	50–65	Normal saline Hypertonic saline Fresh frozen plasma	14–63	110 62 70	MAP, CO, HR, Hb, DO_2_
2	Sondeen et al. (2011)	5	8	38.7 (1.9)	150	44–50	Lactated Ringer’s solution Fresh frozen plasma Fresh whole blood Plasma-PRBC (1:1) Plasma-PRBC (1:4)	21–63	98 63 60 55 68	MAP, CO, HR, Hb, DO_2_
3	Urbano et al. (2012)	3	11 or 12	9.8 (2.0)	150	31	Normal saline Hypertonic saline Albumin + hypertonic saline	15–31	52 26 26	MAP, CI, HR, SvO_2_
4	(Unpublished data)^c^	9	11 or 12	40.3 (2.4)	80	50–79	Normal saline Fresh frozen plasma Fresh whole blood	15–63	26–315 21–196 9–196	MAP, CO, HR, Hb, DO_2_

CI, cardiac index; CO, cardiac output; DO_2_, delivered oxygen; Hb, hemoglobin; HR, heart rate; MAP, mean arterial pressure; PRBC, packed red blood cells; SvO_2_, mixed venous blood oxygen saturation.

^a^ Presented as mean (standard deviation).

^b^ MAP and CO data used to calibrate the model.

^c^ Each fluid was provided at three different resuscitation rates (slow, standard, or bolus infusion), for each range.

#### 2.2.1 Model calibration

To ensure that the extended CR model accurately captured the changes in vital-sign response to hemorrhage and resuscitation fluid type, we calibrated 13 of the 118 model parameters using the swine study data reported by Soller et al. (2014) in *Study 1*. To identify the parameters that needed to be calibrated, we performed a local sensitivity analysis and identified five parameters of the transcapillary fluid shift component whose changes had the greatest effect on MAP following hemorrhage. In addition, because the transcapillary fluid shift was one of the cardiovascular regulatory mechanisms activated during hemorrhage and worked in tandem with other control loops present in the CR model, we fine-tuned eight control parameters of the original CR model, as well. For eight of the 13 parameters, we defined their feasible ranges using available literature data (Guyton, 1980; Mazzoni et al., 1988; Michel, 1988; Just et al., 1998; Elstad et al., 2002; Pstras and Waniewski, 2019), and for the other parameters whose ranges were not available in the literature, we used ±60% of their nominal values to define their feasible range.

For model calibration, we used the MAP and CO data reported in *Study 1,* where animals were bled to ∼45% of their total blood volume followed by resuscitation using three distinct fluid types (NS, HTS, and FFP). Although *Study 1* also reported HR data (a vital sign predicted by the CR model), we did not use these data for model calibration because HR dynamics are highly variable and affected by a variety of factors, such as pain and stress, which we do not currently account for in the model (Jin et al., 2023). Because the experiments were performed on animals and the CR model represents a human, we normalized the inputs before simulating the scenarios. Based on the correlation defined by Watts et al. (2023), we used the average body weight of the animals in each study to compute the corresponding hemorrhage and resuscitation rates as a percentage of the total blood volume. We then multiplied these percentages by the blood volume of an adult (70 kg, 5 L blood) to identify the equivalent volumetric hemorrhage and resuscitation rates for a human adult and used them as inputs to the model. Similarly, we also normalized the predicted outputs (MAP and CO) because their values at the baseline conditions varied between the different experiments as well as between the model simulations. Accordingly, we multiplied each simulated output by the ratio of the baseline experimental value (group mean) to the baseline simulated value of the corresponding model output (Jin et al., 2023).

To identify the optimal parameter values, we minimized the root mean square error (RMSE) between the experimental data and the model-predicted MAP and CO. Because MAP and CO represent distinct quantities with different units, we scaled the RMSE values of each output with their respective standard error of the mean (SEM). We defined the model’s “nominal parameter set” as the final parameter values obtained after performing the calibration procedure (Supplementary Table S1).

In addition, we assumed that in the absence of hemorrhage or fluid resuscitation, the fluid exchange model was in dynamic equilibrium (Xie et al., 1995; Rosalina et al., 2019). Based on this assumption, we calculated the initial values of the lymphatic flow rate and the concentrations of salt and protein in all five compartments by solving the equations in the transcapillary fluid exchange component of the model (Equations 4–11) after setting the net transport of albumin, salt, and water to zero.

#### 2.2.2 Model validation

To validate the extended model, we compared its predictions against experimental data from three existing studies (*Studies 2*-*4* in Table 1). Briefly, these studies involved swine models challenged with hemorrhage ranging from 31%–79% of total blood volume and resuscitation using six different fluid types, including NS, HTS, FFP, LR, FWB, and PRBC, administered individually or in combination. *Study 3* consisted of a single phase of controlled hemorrhage followed by fluid resuscitation. In contrast, *Studies 2* and *4* consisted of both controlled and uncontrolled hemorrhage, which in turn resulted in a re-bleed phase following fluid resuscitation. In addition, *Study 4* involved fluid resuscitation at three different infusion rates (slow, standard, and bolus infusion) for each fluid type. The studies reported vital signs, blood variables, and the rates of hemorrhage and resuscitation. For additional information on the experimental protocols, we refer the reader to the original articles (Sondeen et al., 2011; Urbano et al., 2012).

To replicate the scenarios in *Studies 2-4 in silico,* we provided the corresponding hemorrhage, fluid resuscitation, and ventilation rates as inputs to the model, after normalizing them (and the outputs) based on the procedure described in Section 2.2.1 “Model calibration.” To validate the model, we compared the changes in the time course of the various model outputs (MAP, CO, HR, Hb, DO_2_, and SvO_2_) with their corresponding experimental measurements by computing the RMSE (Table 2).

**TABLE 2:** Root mean square error (RMSE) between model-predicted vital signs and blood variables and the corresponding experimental data.

Study	Fluid type/Infusion rate		RMSE					
			MAP (mmHg)	CO (L/min)	HR (beats/min)	Hb (g/dL)	DO_2_ [ml/(kg·min)]	SvO_2_ (%)
1	Normal saline		8.85^a^	0.41^a^	40.03	0.64	0.88	-
	Hypertonic saline		12.31^a^	0.76^a^	49.90	0.82	1.31	-
	Fresh frozen plasma		8.59^a^	0.45^a^	31.20	0.78	1.56	-
2	Lactated Ringer’s solution		6.76	0.38	33.73	0.74	1.30	-
	Fresh frozen plasma		5.60	0.42	52.26	0.92	0.88	-
	Fresh whole blood		3.38	0.40	57.89	1.09	1.27	-
	Plasma-PRBC (1:1)		2.48	0.41	50.63	1.69	0.67	-
	Plasma-PRBC (1:4)		2.18	0.29	72.48	1.64	0.80	-
3	Normal saline		5.53	0.87^b^	41.06	-	-	5.28
	Hypertonic saline		11.14	0.57^b^	42.65	-	-	8.12
	Albumin + hypertonic saline		4.20	0.78^b^	24.50	-	-	7.07
4	Normal saline	Standard infusion	8.61	0.48	49.83	0.40	0.58	-
		Slow infusion	11.47	0.57	37.24	0.42	0.60	-
		Bolus infusion	6.48	0.53	49.75	0.45	0.71	-
	Fresh frozen plasma	Standard infusion	8.53	0.57	31.05	0.29	0.92	-
		Slow infusion	7.15	0.46	44.20	0.27	0.35	-
		Bolus infusion	10.16	0.46	36.51	0.40	0.73	-
	Fresh whole blood	Standard infusion	9.22	0.38	37.71	0.65	0.47	-
		Slow infusion	6.88	0.33	48.60	0.76	0.24	-
		Bolus infusion	7.72	0.39	46.67	0.32	0.33	-
Average RMSE, *Studies 2*–*4*			6.91	0.49	44.52	0.72	0.70	6.82

CO, cardiac output; DO_2_, delivered oxygen; Hb, hemoglobin concentration; HR, heart rate; MAP, mean arterial pressure; PRBC, packed red blood cells; SvO_2_, mixed venous blood oxygen saturation.

^a^ Data used to calibrate the model.

^b^ RMSE for cardiac index (L/min/m^2^).

### 2.3 *In silico* analysis of different fluid resuscitation treatments

We used the validated extended CR model to investigate the cardiovascular dynamics caused by changes in blood volume and in DO_2_ to the tissues during different resuscitation treatment scenarios. Specifically, we performed three simulations. In the first, we analyzed the effect of changes in blood volume on the cardiovascular dynamics by simulating a total hemorrhage of 2.75 L, using the nominal parameter set. We introduced the hemorrhage in four consecutive 10-min intervals followed by three consecutive 5-min intervals, for a total of seven hemorrhage intervals with a constant bleeding rate of 50 mL/min. Between each of the seven bleeding intervals, we added six 30-min non-bleeding periods so that we could obtain CO and blood volume values after the model had achieved steady state. In these simulations, we kept the Hb concentration at a constant value of 12.8 g/dL. In addition, at the end of each of the seven hemorrhage intervals, we computed the time required by the blood to complete one-half of the body’s circulation, i.e., the lung-to-periphery circulation time.

In the second simulation, we investigated through four scenarios the effect of resuscitation with different fluid types on DO_2_ to the tissues. Each scenario included hemorrhage of 40% of the total blood volume over a 30-min interval followed by a 30-min non-bleeding period. The first two scenarios involved resuscitating with FWB after the non-bleeding period at a rate equal to that of hemorrhage until the total infusion volume reached either 40% or 32% of the initial blood volume. We repeated the same two simulations using FFP as the resuscitating fluid. We chose to evaluate the effect of FWB and FFP because they are two of the most widely recommended fluids for hemorrhagic trauma (Holcomb et al., 2007). We compared the time course of changes in DO_2_ between the four scenarios in an attempt to differentiate between the two fluids’ ability to transport oxygen to the tissues.

Finally, in the third simulation, we assessed a wide range of hemorrhage scenarios (10%–35% of blood volume) with resuscitation treatments involving distinct fluid types (NS, FFP, FWB, and PRBC). This included a 30-min hemorrhage period followed by a 30-min non-bleeding period, followed by a 30-min resuscitation period. In each of the hemorrhage and fluid-treatment scenarios, we computed the minimum volume of resuscitation fluid required to restore DO_2_ to 80% of its baseline value, while using an upper bound of 3 L (60% of blood volume) as the maximum amount of fluid that could be infused.

## 3 Results

### 3.1 Model calibration

Based on the local sensitivity analysis, we identified five parameters of the transcapillary fluid exchange component for calibration, including the hydraulic conductivity $L_{\text{Pl}‐\text{ISS}}$ , the reflection coefficient of salt $\sigma_{\text{Pl}‐\text{ISS}}^{\text{salt}}$ , the permeability-surface area product of albumin $PS_{\text{Pl}‐\text{ISS}}^{\text{alb}}$ , the upper bound on the transcapillary refill $V_{\text{refill}}^{\max}$ , and the coefficient k_cp_ used for calculating the hydrostatic pressure in the capillaries. We calibrated the values of these parameters, along with eight parameters from the original CR model (Supplementary Table S1). We verified that after calibration the estimated values of two key model parameters, the product of the surface area and hydraulic conductivity of the plasma-ISS boundary and the Peclet number for albumin transport along the same boundary, were similar to those identified by other computational models [ 6.5 mL/(min·mmHg) vs. 5.0 to 9.0 mL/(min·mmHg) and 0.03 vs. 0.01 to 0.07, respectively (Tatara et al., 2007; Pstras and Waniewski, 2019; Rosalina et al., 2019)].

**FIGURE 2:**
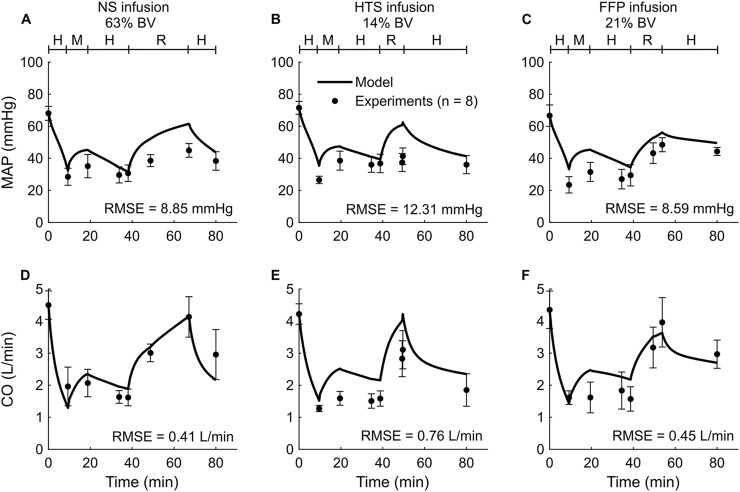
Comparison of the experimental vital-sign data from *Study 1* and the corresponding model fits. **(A-C)** Mean arterial pressure (MAP) and **(D-F)** cardiac output (CO). The left panels show the results for infusion of normal saline (NS), the middle panels for 3% hypertonic saline (HTS), and the right panels for fresh frozen plasma (FFP). The timeline at the top of each panel illustrates the hemorrhagic shock protocol, wherein H represents the hemorrhage period, M denotes the monitoring period, and R represents the resuscitation period. The error bars denote two standard errors of the mean. BV: blood volume; RMSE: root mean square error.

We used the experimental data reported in *Study 1* to calibrate the parameters of the extended CR model. The study reported changes in MAP and CO of swine studies performed for three distinct experimental groups, with each group using a different resuscitation fluid type (NS, HTS, or FFP), following hemorrhage of ∼45% of their total blood volume. Figure 2 shows the calibrated (solid line) and experimentally measured (filled circles) MAP and CO data, which indicate similar trends and a reasonable agreement. For example, both values decreased by ∼55% of their nominal values during hemorrhage and returned to ∼80% after resuscitation. The results indicate that the model captured the general trend of experimental data with RMSEs smaller than 12.31 mmHg for MAP and 0.76 L/min for CO. Table 2 shows the individual RMSEs for each of the three experimental groups and the error for other vital signs (HR) and blood variables (Hb and DO_2_), which we did not use for model calibration. Supplementary Figures S1A–C in the Supplementary Material show the simulated (solid line) and experimentally measured (filled circles) HR data.

### 3.2 Model validation

**FIGURE 3:**
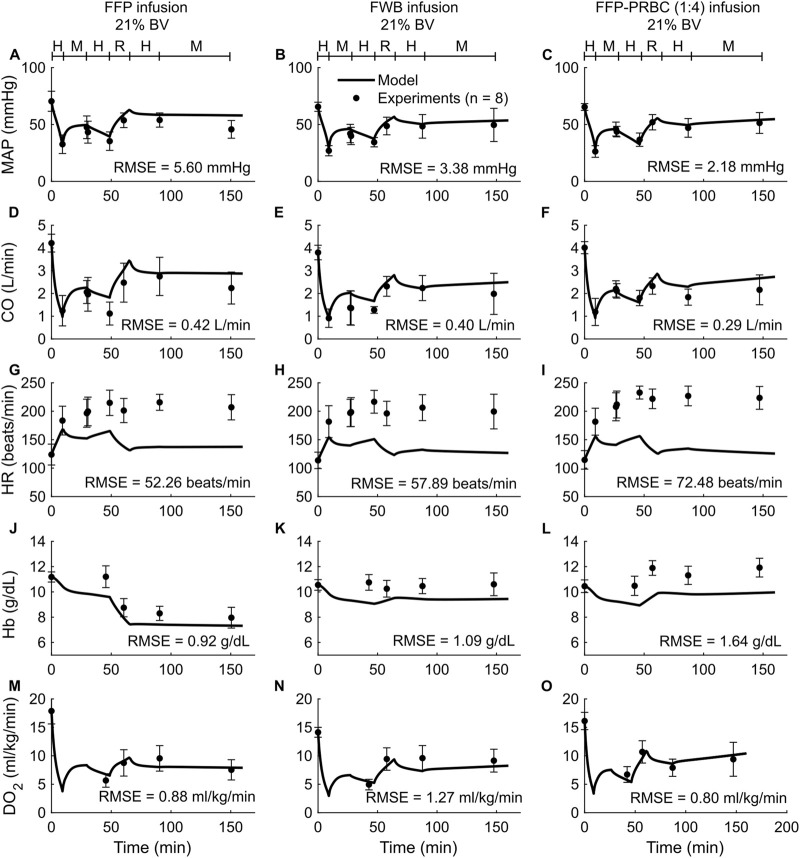
Comparison of model predictions of vital signs and blood variables from *Study 2* and the corresponding experimental data. **(A–C)** Mean arterial pressure (MAP), **(D–F)** cardiac output (CO), **(G–I)** heart rate (HR), **(J–L)** hemoglobin concentration (Hb), and **(M–O)** delivered oxygen (DO_2_). The left panels show the results for infusion of fresh frozen plasma (FFP), the middle panels for fresh whole blood (FWB), and the right panels for FFP and packed red blood cells (PRBC) in a 1:4 ratio [FFP-PRBC (1:4)]. The timeline at the top of each panel illustrates the hemorrhagic shock protocol, wherein H represents the hemorrhage period, M denotes the monitoring period, and R represents the resuscitation period. The error bars denote two standard errors of the mean. BV: blood volume; RMSE: root mean square error.

To validate the computational model, we compared its predictions of the time course of changes in vital signs (MAP, CO, and HR) and blood variables (Hb, DO_2_, and SvO_2_) with the corresponding experimental data from *Studies 2-4* in Table 1. *Study 2* reported changes in MAP, CO, HR, Hb, and DO_2_ across five distinct experimental groups, with each group using a different fluid type [LR, FFP, FWB, FFP-PRBC (1:1), or FFP-PRBC (1:4)] for resuscitation following hemorrhage. For brevity, we only show the comparisons for three out of the five groups, but we provided the RMSEs for all five groups in Table 2. Figure 3 shows the model predictions (solid lines) for MAP, CO, HR, Hb, and DO_2_ and the corresponding experimental data (filled circles). The predictions for MAP and CO showed reasonable agreement with the experimental data, with nearly 90% of the predictions falling within 2 SEM of the experimental data. However, for HR, we observed considerable differences between the model predictions and the experimental data (10% of the model predictions fell within 2 SEM of the experimental data; average RMSE = 53 beats/min. For Hb and DO_2_, 54% of the model predictions fell within 2 SEM.

In both the experimental data and our simulations, we only observed modest differences in the changes in the vital signs (MAP, CO, and HR) across the five different types of resuscitation fluid. However, Hb was sensitive to fluid type. For example, for the same degree of hemorrhage in *Study 2*, the concentration of Hb decreased by 21% after FFP infusion but increased by 11% after FFP-PRBC (1:4) infusion, which the model captured (Figures 3J,L). However, during hemorrhage (the first 50 min of the simulation), the model predicted a 14% drop in Hb concentration, which was not observed in the experiments. Finally, we observed that fluid resuscitation always resulted in an increase in DO_2_, regardless of fluid type (Figures 3M–O).

**FIGURE 4:**
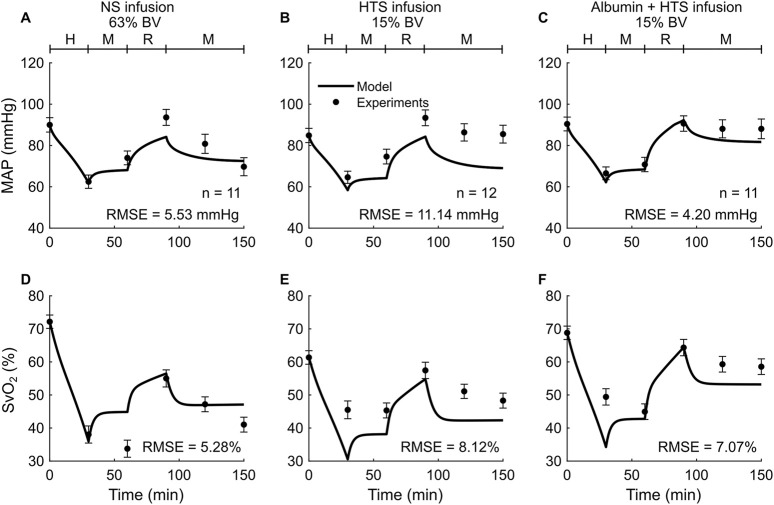
Comparison of model predictions of a vital sign and a blood variable from *Study 3* and the corresponding experimental data. **(A-C)** Mean arterial pressure (MAP) and **(D-F)** mixed venous blood oxygen saturation (SvO_2_). The left panels show the results for infusion of normal saline (NS), the middle panels for 3% hypertonic saline (HTS), and the right panels for 5% albumin plus 3% hypertonic saline (Albumin + HTS). The timeline at the top of each panel illustrates the hemorrhagic shock protocol, wherein H represents the hemorrhage period, M denotes the monitoring period, and R represents the resuscitation period. The error bars denote two standard errors of the mean. BV: blood volume; RMSE: root mean square error.

*Study 3* reported changes in four outputs (HR, MAP, cardiac index, and SvO_2_) across three distinct experimental groups, with each group using a different resuscitation fluid type (NS, HTS, or Albumin + HTS) following hemorrhage. For brevity, we only show the comparisons for two (MAP and SvO_2_) of the four outputs (Figure 4), with the other results reported in Table 2 and Supplementary Figures S1D–F (Supplementary Material). In general, the predictions for both MAP and SvO_2_ showed reasonable agreement with the experimental data, with 33% of the predictions falling within 2 SEM. For each of the three groups, we observed considerable differences in the experimentally measured values of MAP (∼79%–88% of their baseline values; Figures 4A–C, filled circles) and SvO_2_ (∼47%–74% of their baseline values; Figures 4D–F, filled circles) for the same hemorrhagic injury before the start of the resuscitation, which might have contributed large RMSEs between the model predictions and the mean experimental values.

**FIGURE 5:**
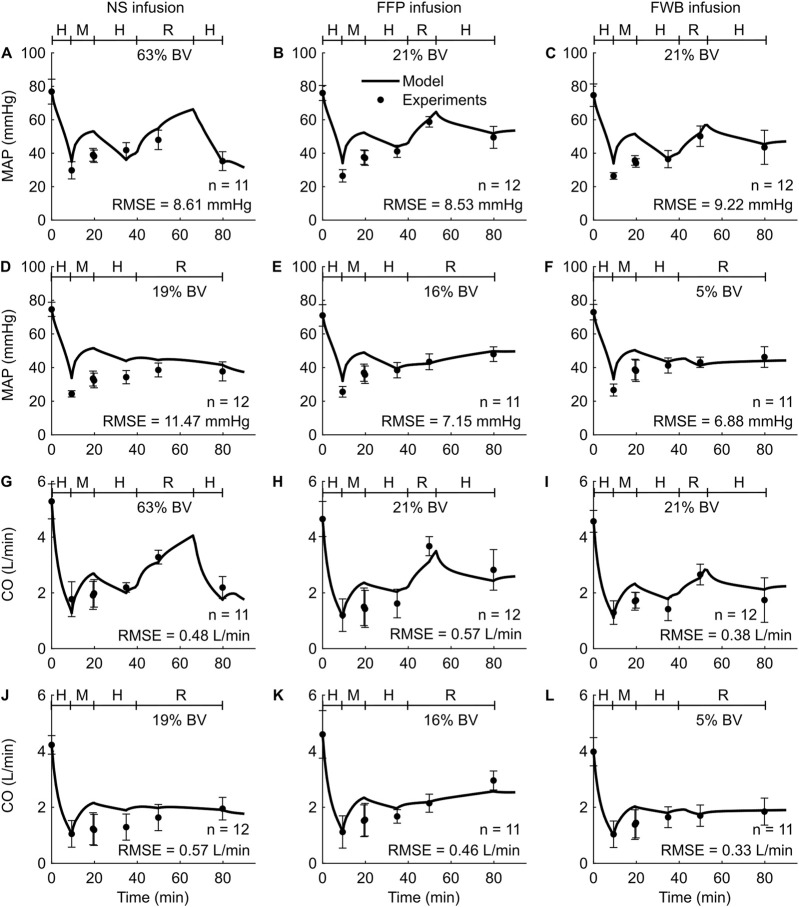
Comparison of model predictions of vital signs from *Study 4* and the corresponding experimental data. **(A-C)** Mean arterial pressure (MAP) for standard infusion, **(D-F)** MAP for slow infusion, **(G-I)** cardiac output (CO) for standard infusion, and **(J-L)** CO for slow infusion. The left panels show the results for infusion of normal saline (NS), the middle panels for fresh frozen plasma (FFP), and the right panels for fresh whole blood (FWB). The timeline at the top each subplot illustrates the hemorrhagic shock protocol, wherein H represents the hemorrhage period, M denotes the monitoring period, and R represents the resuscitation period. The error bars denote two standard errors of the mean. BV: blood volume; RMSE: root mean square error.

*Study 4* investigated the effects of three fluid types (NS, FFP, and FWB), where each fluid was administered at each of three different resuscitation rates (slow, standard, and bolus infusion), and reported changes in five outputs (HR, MAP, CO, Hb, and DO_2_) across the nine distinct experimental groups. For brevity, Figure 5 only shows the comparisons for two outputs (MAP and CO) from six experimental groups (slow or standard infusion for each of the three different fluid types). We show the HR comparisons for each of the six groups in Supplementary Figure S2 (Supplementary Material) and all the RMSEs in Table 2. In both the experimental data and our simulations, we observed that when different resuscitation fluids were transfused at their standard infusion rates [1.7 mL/(kg·min) for NS and 1.2 mL/(kg·min) for both FFP and FWB], resuscitation resulted in MAP returning to ∼75% of its nominal value. However, after resuscitation, we observed that NS resulted in a sharp drop in MAP in the absence of additional active hemorrhage (Figures 5A–C). In contrast, when these fluids were infused at a slower rate [0.4 mL/(kg·min) for NS, 0.3 mL/(kg·min) for FFP, and 0.1 mL/(kg·min) for FWB], resuscitation by all fluid types resulted in a smaller recovery in MAP (returning to ∼60% of its nominal value). However, for all infusions, MAP remained relatively stable after resuscitation (Figures 5D–F). In these comparisons, on average, 49% of the model predictions for MAP and CO fell within 2 SEM.

Overall, for the validation studies (*Studies 2–4*), we observed that the experimental data and the model predictions demonstrated similar trends, i.e., as expected the values of MAP, CO, DO_2_, and SvO_2_ decreased during hemorrhage and increased during fluid resuscitation (regardless of fluid type), while the values for HR increased during hemorrhage and decreased during fluid resuscitation. In contrast, changes in Hb concentration depended on the resuscitation fluid type. Furthermore, the RMSEs between the different model outputs and the corresponding experimental data were in ranges similar to those obtained during model calibration with *Study 1* (Table 2).

### 3.3 Simulation results

#### 3.3.1 Oxygen delivery increased with fluid resuscitation

**FIGURE 6:**
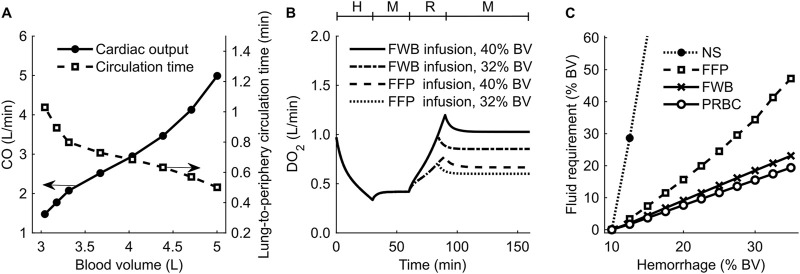
Results of simulations performed to investigate changes in blood volume (BV) and delivered oxygen (DO_2_) for different resuscitation treatment scenarios. **(A)** Cardiac output (CO) and blood circulation time as a function of BV in the body. **(B)** DO_2_ during hemorrhage followed by resuscitation using fresh whole blood (FWB) or fresh frozen plasma (FFP). **(C)** Fluid required to achieve a final DO_2_ of 80% of its original value. The timeline at the top of **(B)** illustrates the hemorrhagic shock protocol, wherein H represents the hemorrhage period, M denotes the monitoring period, and R represents the resuscitation period. NS: normal saline; PRBC: packed red blood cells.

To investigate the effect of changes in blood volume on cardiovascular dynamics, we simulated a scenario of progressive hypovolemia, i.e., a blood loss that increased from 0% to 55% of the initial blood volume. Figure 6A shows the variations in CO (filled circles) and the lung-to-periphery circulation time (open squares) as a function of blood volume in the body. As expected, we observed that CO monotonically increased and that lung-to-periphery circulation time monotonically decreased with an increase in blood volume.

Next, we investigated the effect of different resuscitation fluid types on DO_2_. Figure 6B shows the model-generated changes in DO_2_ following hemorrhage and resuscitation with two different fluids: FWB (with Hb; solid line and dashed-dotted line) or FFP (without Hb; dashed line and dotted line). At the end of hemorrhage (40% of blood volume between 0 and 30 min), DO_2_ decreased to 33% of its nominal value. At the end of fluid resuscitation (40% of blood volume between 60 and 90 min), DO_2_ returned to 100% of its original value when we used FWB as the resuscitation fluid but to only 67% when we used FFP. We observed a similar trend when we used a lower resuscitation volume (32% blood volume), but the corresponding recovery in DO_2_ was also lower (85% for FWB and 60% for FFP).

#### 3.3.2 Volume of resuscitation fluid required to restore oxygen delivery varied with the fluid type

We performed simulations to determine the infusion volume of different resuscitation fluids required to meet a treatment target, i.e., to restore DO_2_ to 80% of its original value following hemorrhage ranging from 10% to 35% of total blood volume. Figure 6C shows the predicted volumes required to achieve the DO_2_ target for different resuscitation fluids (i.e., NS, FFP, FWB, or PRBC). Based on these results, we found that when resuscitating with PRBC (Figure 6C, open circles) or FWB (Figure 6C, crosses), the amount of fluid volume required to meet the target increased linearly with hemorrhage severity. However, compared with FWB, resuscitation with FFP (Figure 6C, squares) required 65%–100% more fluid, and the slope of the curve increased with hemorrhage severity. Finally, we found that, even for a mild hemorrhage scenario (13% of blood volume), resuscitation with NS (Figure 6C, filled circles) required fluid volumes greater than 10 times those required for FWB to meet the same DO_2_ target. In addition, in the simulations where hemorrhage resulted in a total blood loss greater than 13% of blood volume, we found that even when we used 3 L of NS infusion, the resuscitation failed to restore DO_2_ to 80% of its nominal value.

## 4 Discussion

Given the numerous challenges combat medics will face during prolonged casualty care in LSCO, including high casualty rates, large casualty-to-provider ratios in a resource-constrained environment, and delayed evacuation, AI technologies that optimize and accelerate decision-making on the battlefield will become increasingly essential to aid combat medics in the delivery of care (Jin et al., 2023). The development of such technologies requires accurate predictive models that can reliably reproduce the physiological responses to major battlefield injuries, in particular hemorrhage, the leading cause of preventable death on the battlefield (Eastridge et al., 2012; Kotwal et al., 2018; Eastridge et al., 2019). To address this gap, we previously developed and validated the CR model and used it to generate synthetic data of hemorrhagic trauma patients and resuscitation treatments using a nonspecific fluid type that only considered changes in the volume of the infused fluid (Jin et al., 2023). We subsequently used the synthetic data to develop and assess AI algorithms that optimize fluid utilization in resource-constrained mass-casualty scenarios (Jin et al., 2024). Here, we extended the CR model by incorporating the effects of six different types of resuscitation fluids (NS, LR, HTS, FFP, PRBC, and FWB) on vital signs (MAP, CO, and HR) and blood variables (blood volume, Hb, DO_2_, and SvO_2_), and validated the results using three distinct experimental studies. In addition to volumetric changes, each fluid accounted for its oncotic pressure, which depends on the concentration of proteins and salt in the fluid, thus enhancing the capability of the CR model to simulate a broader range of treatment scenarios following hemorrhage.

We assessed the performance of the extended CR model by comparing its predictions with experimental data reported by three different studies, involving a total of six different types of resuscitation fluids in various hemorrhage scenarios. On average, the RMSEs between the model predictions and the corresponding experimental values for MAP (RMSE = 6.91 mmHg), CO (0.49 L/min), Hb (0.72 g/dL), DO_2_ [0.70 mL/(kg·min)], and SvO_2_ (6.82 mmHg) were between 5% and 11% of the average values in humans (Hall and Hall, 2020). However, not surprisingly, the model predictions for HR showed substantial differences from the experimental data (the RMSE varied from 25 to 72 beats/min). Accurate prediction of HR is notoriously challenging during transient conditions, as previously observed in our own work (Jin et al., 2023) as well as in other modeling efforts (Kanal et al., 2022). A potential reason for these differences is that HR dynamics are affected by several factors, such as pain and distress, that we currently do not account for in the CR model due to a lack of understanding of their underlying mechanisms (Tousignant-Laflamme et al., 2005; Jin et al., 2023). In the future, conducting a prospective validation study, where we make predictions and then perform experiments to validate the model predictions, would further substantiate the reliability and generalizability of the model predictions.

A primary goal of fluid resuscitation after hemorrhage is to recover and maintain blood pressure in the arteries by increasing blood volume (Watts, 1998; Kuo and Palmer, 2022). We observed that, after resuscitation, the amount of blood volume expansion varied with fluid type. For example, as reported in *Studies 1* and *4* (Figures 2A,C, 5A–C), during the resuscitation phase, the volume of NS required to maintain MAP was three times greater than that of FFP or FWB. Unlike these fluids, when resuscitating with NS, a substantial portion of the fluid moved from the capillaries into the ISS, requiring a larger volume of NS to restore MAP to the same levels. Our model simulations showed that this difference arose due to the absence of plasma proteins (large molecules) in NS. These proteins, which are present in blood and blood products, exert an oncotic pressure that prevents the fluid from moving from the capillaries into the ISS. It is noteworthy that in *Studies 1* and *4*, for the same reason (i.e., absence of plasma proteins), the post-resuscitation decrease in MAP was also much greater when NS was used as the infusion fluid (∼24% of nominal; Figures 2A, 5A) compared with those observed using FFP or FWB (∼10% of nominal; Figures 2C, 5B,C).

In addition to maintaining blood pressure, a key goal of any fluid resuscitation treatment is to maintain DO_2_ (Kuo and Palmer, 2022), which is the product of CO and arterial oxygen concentration. During resuscitation with fluids that do not contain Hb (e.g., NS, LR, or FFP), even though CO increases as a result of an increase in blood volume, the simultaneous hemodilution can lead to a decrease in arterial oxygen concentration (Siam et al., 2014; Quispe-Cornejo et al., 2022). The net change in DO_2_, therefore, depends on the magnitude of these two counteracting effects. Except for a few studies that show a reduction in oxygen delivery locally in specific tissues in the body [e.g., cerebral (Prough et al., 1986) or renal (Wan et al., 2007)], in general, it is expected that the increase in CO during fluid resuscitation surpasses the decrease caused by hemodilution, resulting in a net increase in DO_2_ (Quispe-Cornejo et al., 2022). Our results corroborate this effect because we also observed that as CO increased with increases in blood volume, the lung-to-periphery circulation time monotonically decreased (Figure 6A, open squares). Hence, during fluid resuscitation, even if the infused fluid (e.g., NS) introduced no additional Hb molecules, the mere expansion of the blood volume enabled the available Hb molecules to circulate through the cardiovascular system faster, achieving a higher DO_2_ compared with its pre-resuscitation value.

Computational models have the potential to augment decision-making in trauma care. For example, choosing the appropriate combination of resuscitation fluid type and volume with which to treat mass casualties, especially when resources are limited, is both crucial and challenging (Jin et al., 2024). In such situations, computational models could be used to identify the optimal treatment strategy for different scenarios. To illustrate this, using the nominal model representing a single “average” casualty, we performed a set of simulations to identify the volume of different fluid types required to meet a given treatment target (i.e., returning DO_2_ after hemorrhage to at least 80% of its nominal value). For mild hemorrhage, we found that the volumes of FFP or NS required to restore DO_2_ were, in general, substantially higher (>1.6 times) than those of FWB or PRBC. However, for moderate or severe hemorrhage (i.e., hemorrhage of >13% blood volume), the FFP volume needed increased to 2 times that of FWB or PRBC, and it was not feasible to restore DO_2_ using NS (Figure 6C), even when we used 3 L of resuscitation fluid. Because neither NS nor FFP contains Hb, their use leads to dilution of Hb in the blood and higher volume requirements to maintain the same DO_2_ level compared with FWB or PRBC. In addition, as discussed above, the loss of fluid from the capillaries when using NS resulted in greater volume requirements to achieve the same DO_2_ target and, in severe hemorrhage, extended beyond the 3-L limit set in our simulation. In the future, we could potentially use the CR model to investigate different treatment targets, such as arterial pressure values, or add constraints on the volume and types of fluid that are available to mimic a resource-constrained environment.

Although by and large the extended CR model captured the variations in vital signs and blood variables during hemorrhage and resuscitation, it does have several limitations due to simplifying assumptions we had to make during model development. First, we calibrated and validated the model using swine data due to the lack of clinical human data. We attempted to overcome this limitation by normalizing the experimental inputs and the model outputs to an average human. Second, while the model captures the majority of the key cardiovascular and respiratory responses to hemorrhagic trauma and fluid resuscitation at the physiological level, it does not account for the cellular and molecular mechanisms arising from hemorrhagic shock, such as coagulopathy, ischemia-reperfusion injury, endothelial cell injury, glycocalyx disruption, and tissue-level CO_2_ retention (Hess et al., 2008; Abassi et al., 2020; Endo et al., 2021). Therefore, the model currently cannot capture the contribution of these mechanisms to changes in the vital signs. Third, the CR model does not account for the effects of pain, anxiety, or medications provided during trauma, because we currently do not have a precise understanding of the mechanisms through which these factors affect vital signs. When such data become available, we will incorporate them in the model to increase its accuracy.

In summary, we extended and validated our previously developed CR model to predict the time course of an individual’s vital-sign changes following hemorrhagic trauma and account for the volumetric and oxygen delivery effects of different resuscitation fluid types. This extension will allow for the generation of a large population of synthetic trauma casualties and the design of optimal fluid allocation strategies for hemorrhagic trauma, considerably expanding treatment options to train and assess the performance of AI algorithms to aid combat medics during prolonged casualty care.

## Data Availability

The original contributions presented in the study are included in the body of the article/Supplementary Material. Further inquiries can be directed to the corresponding author.

## Abbreviations

AI, artificial intelligence; BV, blood volume; CI, cardiac index; CO, cardiac output; CR, cardio-respiratory; DO_2_, delivered oxygen; FFP, fresh frozen plasma; FiO_2_, fraction of inspired oxygen; FWB, fresh whole blood; Hb, hemoglobin; HR, heart rate; HTS, hypertonic saline; ISS, interstitial space; LR, lactated Ringer’s solution; LSCO, large-scale combat operations; MAP, mean arterial pressure; ML, machine learning; MV, minute ventilation; NS, normal saline; PRBC, packed red blood cells; RBC, red blood cells; RMSE, root mean square error; SEM, standard error of the mean; SvO_2_, mixed venous blood oxygen saturation; USAISR, U.S. Army Institute of Surgical Research.
