# Supplementary material for: A model to simulate human cardio-respiratory responses to different fluid resuscitation treatments after hemorrhagic injury
Source: Front Physiol. 2025 Jul 1;16:1613874. doi: 10.3389/fphys.2025.1613874 (PMC12259663; doi:10.3389/fphys.2025.1613874)
Supplement: Supplementary file 1 [file DataSheet1.docx]

***Supplementary Material***

A model to simulate human cardio-respiratory responses to different fluid resuscitation treatments after hemorrhagic injury

Varghese Kurian^1,2^, Xin Jin^1,2^, Sridevi Nagaraja^1,2^, Anders Wallqvist^1^, and Jaques Reifman^1*^

^1^Department of Defense Biotechnology High Performance Computing Software Applications Institute, Defense Health Agency Research & Development, Medical Research and Development Command, Fort Detrick, MD, USA

^2^The Henry M. Jackson Foundation for the Advancement of Military Medicine, Inc., Bethesda, MD, USA

# Text S1. Model Equations of the Extended Cardio-respiratory Model

## Cardiovascular dynamics

We used Guyton’s model of the cardiovascular system with minor modifications (Guyton, 1980). We enforced overall mass balances for the vascular compartments, including the heart, arteries, and veins, to obtain the circulatory dynamics, as shown in Equations (S1)–(S12). Following the convention established by Guyton, we used the following units: volume in liters (L), time in minutes (min), pressure in millimeters of mercury (mmHg), and their derivatives. Any additional units are as defined in the paragraphs where they are used. Variables with an overdot represent the time derivative of the corresponding variable.

We calculated the excess volume of blood in the venous compartment V_ve_ by subtracting the unstressed volume of blood $\text{V}_{\text{vu}}$ from the total volume of the venous compartment, as follows:

| $\text{V}_{\text{ve}}\text{ =}\text{ V}_{\text{v0}}\text{+∫[}\text{f}_{\text{v}}\text{-}\text{f}_{\text{va}}\text{+0.84}\left( \text{h-r} \right)]\text{dt-}\text{V}_{\text{vu}}$ | $\text{(S1)}$ |
| --- | --- |

where V_v0_ denotes the initial blood volume in the veins, f_v_ denotes the blood flow rate from the capillaries to the veins, $\text{f}_{\text{va}}$ denotes the flow rate from the veins to the right atrium, and h and r represent the hemorrhage and fluid resuscitation rates, respectively. We scaled down the hemorrhage and resuscitation rates by a factor of 0.84 because the remaining hemorrhage (~16%) was assumed to change the blood volume in the pulmonary circuit. Given $\text{V}_{\text{ve}}$, we calculated the pressure in the venous compartment $\text{P}_{\text{v}}$ under the assumption of constant venous compliance $\text{C}_{\text{v}}$, as follows:

| $\text{P}_{\text{v}}\text{ = }\text{V}_{\text{ve}}\text{/}\text{C}_{\text{v}}$ | $\text{(S2)}$ |
| --- | --- |

The venous resistance $\text{R}_{\text{v}}$ varied as a function of the venous pressure and the regulation due to blood gas concentrations $\text{δ}$:

| $\text{R}_{\text{v}}\text{ = 3.7}\left( R_{v0}/P_{v} \right)\left( \text{1+}\text{δ}_{\text{resist}} \right)\text{·}\text{δ}_{\text{local}}$ | $\text{(S3)}$ |
| --- | --- |

where $\text{δ}_{\text{resist}}$ denotes the sympathetic effects and $\text{δ}_{\text{local}}$ denotes the local regulation due to changes in blood gas concentrations. As P_v_ has a nominal value of 3.7 mmHg, the gain of 3.7 essentially represents a normalization factor. We calculated the net accumulation rate of blood in the right atrium $\text{f}_{\text{ra}}$ by subtracting the outflow rate from the inflow rate:

| $\text{f}_{\text{ra}}\text{ = }\text{f}_{\text{va}}\text{-CO = }\left[ \left( \text{P}_{\text{v}}\text{-}\text{P}_{\text{ra}} \right)\text{/}\text{R}_{\text{v}} \right]\text{-CO}$ | $\text{(S4)}$ |
| --- | --- |

where P_ra_ denotes the blood pressure of the right atrium and CO denotes the cardiac output. We computed the excess volume of blood in the right atrium $\text{V}_{\text{rae}}$ by subtracting the unstressed blood volume in the right atrium $\text{V}_{\text{rau}}$ from its total volume, as follows:

| $\text{V}_{\text{rae}}\text{ = }\text{V}_{\text{ra0}}\text{+∫}\text{f}_{\text{ra}}\text{dt-}\text{V}_{\text{rau}}$ | $\text{(S5)}$ |
| --- | --- |

where V_ra0_ represents the initial volume of blood in the right atrium. We calculated the pressure in the right atrium under the assumption of constant atrial compliance $\text{C}_{\text{ra}}$:

| $\text{P}_{\text{ra}}\text{ = }\text{V}_{\text{rae}}\text{/}\text{C}_{\text{ra}}$ | $\text{(S6)}$ |
| --- | --- |

In the absence of any controller, we assumed the normal blood flow rate from the heart into the arteries $\text{f}_{\text{an}}$ to be a function of $\text{P}_{\text{ra}}$. We defined the relation between these two variables using cubic splines (fit using the MATLAB function *spline*) connecting the data given by Guyton (1980). The data were originally derived from experiments performed in dogs but were later extrapolated to humans (Jin et al., 2023). We calculated the cardiac output CO by multiplying $\text{f}_{\text{an}}$ with the heart strength HS. Under normal conditions, HS equaled one, but its value could be changed by the cardiovascular controllers as described later in Equation (S46).

We calculated the rate of accumulation of blood in the arteries $\text{f}_{\text{a}}$ by subtracting the outflow ($\text{f}\text{c}$, flow rate into the capillaries) from the inflow (CO), as follows:

| $\text{f}_{\text{a}}\text{ = CO-f}\text{c }\text{= [HS}\text{·}\text{f}_{\text{an}}]\text{-}\text{f}_{\text{c}}$ | $\text{(S7)}$ |
| --- | --- |

We calculated the excess volume V_ae_ of blood in the arteries by subtracting the unstressed volume $\text{V}_{\text{au}}$ of blood from the arterial volume:

| $\text{V}_{\text{ae}}\text{ =}\text{ V}_{\text{a0}}\text{+∫}\text{f}_{\text{a}}\text{dt-}\text{V}_{\text{au}}$ | $\text{(S8)}$ |
| --- | --- |

where $\text{V}_{\text{a0}}$ denotes the initial blood volume in the arteries. We calculated the mean arterial pressure MAP using $\text{V}_{\text{ae}}$ under the assumption of constant arterial compliance $\text{C}_{\text{a}}$:

| $\text{MAP = }\text{V}_{\text{ae}}\text{/}\text{C}_{\text{a}}$ | $\text{(S9)}$ |
| --- | --- |

We calculated the blood flow rate into the capillaries $\text{f}_{\text{c}}$ from the arterial and venous pressures and the arterial resistance $\text{R}_{\text{a}}$:

| $\text{f}_{\text{c}}\text{ = (MAP-}\text{P}_{\text{v}}\text{)/}\text{R}_{\text{a}}$ | $\text{(S10)}$ |
| --- | --- |

We calculated the pressure in the capillaries P_Cap_ from the pressure in the venous compartment and the capillary flow rate, as follows:

| $\text{P}_{\text{Cap}}\text{ = }{\text{P}_{\text{v}}\text{+}\text{k}\text{cp}\text{·}\text{f}}_{\text{c}}$ | $\text{(S11)}$ |
| --- | --- |

where $\text{k}\text{cp}$ denotes a parameter used to calculate the pressure loss in circulation. We computed the net flow rate of blood from the capillaries to the veins $\text{f}_{\text{v}}$ after accounting for the effects of urinary output $\text{f}_{\text{u}}$ and any exchange with the interstitial space ISS, where we obtained the latter from the fluid exchange model described in the main text:

| $\text{f}_{\text{v}}\text{ = }\text{f}_{\text{c}}\text{+0.84(-}\text{J}_{\text{Cap-ISS}}^{\text{v'}}\text{-}\text{J}_{\text{Cap-EC}}^{\text{v'}}\text{+}\text{J}_{\text{L}}^{\text{v}}\text{-}\text{f}_{\text{u}}\text{)}$ | $\text{(S12)}$ |
| --- | --- |

We enforced the mass balance to calculate the volumes of other fluid compartments:

| ${\text{V}_{\text{ISS}}\text{ = V}}_{\text{ISS0}}\text{+∫}\left( \text{J}_{\text{Cap-ISS}}^{\text{v'}}\text{+}\text{J}_{\text{EC-ISS}}^{\text{v'}}\text{-}\text{J}_{\text{ISS-Tcell}}^{\text{v}}\text{-}\text{J}_{\text{L}}^{\text{v}} \right)\text{dt}$ | $\text{(S13)}$ |
| --- | --- |
| $\text{V}_{\text{EC}}= \text{V}_{\text{EC0}}\text{+∫}\left( \text{J}_{\text{Cap-EC}}^{\text{v'}}\text{-}\text{J}_{\text{EC-ISS}}^{\text{v'}} \right)$ | $\text{(S14)}$ |
| $\text{V}_{\text{Tcell}}= \text{V}_{\text{Tcell0}}\text{+∫}\text{J}_{\text{ISS-Tcell}}^{\text{v}}\text{ dt}$ | $\text{(S15)}$ |
| $\text{V}_{\text{RBC}}= \text{V}_{\text{RBC0}}\text{+∫}\text{J}_{\text{Cap-RBC}}^{\text{v}}\text{ dt}$ | $\text{(S16)}$ |

where $\text{V}_{\text{ISS}}$, $\text{V}_{\text{EC}}$, $\text{V}_{\text{Tcell}}$, and $\text{V}_{\text{RBC}}$ denote the volumes of the ISS, endothelial cells, tissue cells, and red blood cells, respectively. The symbols with a subscript 0 indicate the initial value of the respective volumes. We calculated the total volume of blood in the body BV as the sum of the volumes of arterial and venous compartments and the right atrium, multiplied by a factor:

| $\text{BV = (}\text{V}_{\text{ae}}\text{+}\text{V}_{\text{au}}\text{+}\text{V}_{\text{ve}}\text{+}\text{V}_{\text{vu}}\text{+}\text{V}_{\text{rae}}\text{+}\text{V}_{\text{rau}}\text{)}\text{·}\text{1.19}$ | $\text{(S17)}$ |
| --- | --- |

where we used the factor 1.19 to account for the blood volume in the pulmonary circuit (~16% BV), which we do not explicitly model here. We used mass balance equations to also calculate the salt and protein concentrations of the different compartments $\text{C}$ (g/L):

| $\text{C}_{\text{Pl}}^{\text{glob}}\text{ = }\text{C}_{\text{Pl0}}^{\text{glob}}\text{·}\text{B}\text{V}_{\text{0}}\text{/BV}$ | $\text{(S18)}$ |
| --- | --- |
| $\text{C}_{\text{Pl}}^{\text{alb}}\text{ = }\text{[C}_{\text{Pl0}}^{\text{alb}}\text{·}\text{B}\text{V}_{\text{0}}\text{+}\text{∫}{\text{(}\text{-}\text{J}}_{\text{Pl-ISS}}^{\text{alb}}\text{+}\text{J}_{\text{L}}^{\text{w}}{\text{·}\text{C}}_{\text{ISS}}^{\text{alb}}\text{)dt]/BV}$ | $\text{(S19)}$ |
| $\text{C}_{\text{Pl}}^{\text{salt}}\text{ = }\text{[C}_{\text{Pl0}}^{\text{salt}}\text{·}\text{B}\text{V}_{\text{0}}\text{+}\text{∫}{\text{(}\text{-}\text{J}}_{\text{Pl-ISS}}^{\text{salt}}\text{+}\text{J}_{\text{L}}^{\text{w}}{\text{·}\text{C}}_{\text{ISS}}^{\text{salt}}\text{-}\text{f}_{\text{u}}{\text{·}\text{C}}_{\text{Pl}}^{\text{salt}}\text{)dt]/BV}$ | $\left( \text{S20} \right)$ |
| $\text{C}_{\text{ISS}}^{\text{alb}}\text{ = }\text{[C}_{\text{ISS}}^{\text{alb}}\text{·}\text{V}_{\text{ISS0}}\text{+}\text{∫}\text{(J}_{\text{Pl-ISS}}^{\text{alb}}\text{-}\text{J}_{\text{L}}^{\text{w}}{\text{·}\text{C}}_{\text{ISS}}^{\text{alb}}\text{)dt]/}\text{V}_{\text{ISS}}$ | $\text{(S21)}$ |
| $\text{C}_{\text{ISS}}^{\text{salt}}\text{ =}\text{[C}_{\text{ISS0}}^{\text{salt}}\text{·}\text{V}_{\text{ISS0}}\text{+}\text{∫}\text{(J}_{\text{Pl-ISS}}^{\text{salt}}\text{-}\text{J}_{\text{L}}^{\text{w}}{\text{·}\text{C}}_{\text{ISS}}^{\text{salt}}\text{)dt]/}\text{V}_{\text{ISS}}$ | $\text{(S22)}$ |

where the subscript 0 is used to denote the initial value of the respective variables. The red blood cells, endothelial cells, and tissue cells did not have any solute transport across their boundaries. Hence, we calculated the salt concentrations by dividing the initial salt content by the compartment volume at each time instant. We used a similar equation to calculate the hemoglobin concentration (Hb, g/L):

| $\text{Hb = [}\text{H}\text{b}_{\text{0}}\text{·}\text{B}\text{V}_{\text{0}}\text{+}\text{∫}\left( \text{h}\text{·}\text{Hb-r}\text{·}\text{H}\text{b}_{\text{r}} \right)\text{dt]/BV}$ | $\text{(S23)}$ |
| --- | --- |

where $\text{H}\text{b}_{\text{r}}$ represents the hemoglobin concentration of the resuscitation fluid.

## Cardiovascular pressure regulation

Guyton (1980) included the sympathetic stimulation and the angiotensin mechanism for regulation of arterial pressure. We used the same mechanisms in our model. Equations (S24)–(S41) describe these mechanisms. We calculated the effective arterial pressure acting at the baroreceptor P_ab_ by subtracting the baroreceptor adaptation S_ab_ from the MAP, as follows:

| $\text{P}_{\text{ab}}\text{ = MAP-}\text{S}_{\text{ab}}$ | $\text{(S24)}$ |
| --- | --- |

We calculated the baroreceptor adaptation as follows:

| $\text{S}_{\text{ab}}\text{ = ∫}\left[ \text{(}\text{P}_{\text{ab}}\text{-100)/2000} \right]\text{dt}$ | $\text{(S25)}$ |
| --- | --- |

Guyton computed the baroreceptor signal $\text{S}_{\text{s1}}$ from $\text{P}_{\text{ab}}$. We multiplied $\text{P}_{\text{ab}}$ with a factor $\text{k}_{\text{ab}}$, whose value we estimated during calibration, before computing $\text{S}_{\text{s1}}$.

| $\text{P}_{\text{ab1}}\text{ = }\text{k}_{\text{ab}}\text{·}\text{P}_{\text{ab}}$ | $\text{(S26)}$ |
| --- | --- |

We used a sigmoidal relation similar to the one used by Guyton to calculate $\text{S}_{\text{s1}}$. For this, we defined the $\text{P}_{\text{ab1}}$-$\text{S}_{\text{s1}}$ relation by fitting cubic splines to the data given by Guyton (1980).We calculated the stimulated sympathetic activity $\text{S}_{\text{s}}$ from the baroreceptor signal as follows:

| $\text{S}_{\text{s}}\text{ = 1+∫}[\left( \text{S}_{\text{s1}}\text{-}\text{S}_{\text{s}} \right)\text{/}\text{τ}\text{sym}\text{]dt}$ | $\text{(S27)}$ |
| --- | --- |

where $\text{τ}\text{sym}$ represents the time constant for the delay in development of the effect. We computed the sympathetic stimulation factor $\text{S}_{\text{n}}$ as follows:

| $\text{S}_{\text{n}}\text{ = }\text{k}_{\text{sym}}\text{(}\text{S}_{\text{s}}\text{-1)}$ | $\text{(S28)}$ |
| --- | --- |

where the parameter $\text{k}_{\text{sym}}$ denotes the sensitivity of the sympathetic control system. We then used S_n_ as a stimulus for different segments of the circulation:

| $\text{S}_{\text{V}}\text{ = }\text{k}_{\text{SV}}\text{·}\text{S}_{\text{n}}$  $\text{S}_{\text{R}}\text{ = }\left( \text{k}_{\text{SR}}\text{·}\text{S}_{\text{n}} \right)\text{+1}$  $\text{S}_{\text{HS}}\text{ = }\left( \text{k}_{\text{SHS}}\text{·}\text{S}_{\text{n}} \right)\text{+1}$  $\text{S}_{\text{k}}\text{ = }\text{k}_{\text{Sk}}\text{·}{\text{(}\text{S}_{\text{n}}\text{+1)}}^{\text{0.7}}$ | $\text{(S29)}$  $\text{(S30)}$  $\left( \text{S31} \right)$  $\text{(S32)}$ |
| --- | --- |

where S_V_, S_R_, S_HS_, and S_k_ denote the effect of sympathetic stimulation on the venous unstressed blood volume, arterial resistance, heart strength, and kidney function, respectively. The parameters k_SV_, k_SR_, k_SHS_, and k_Sk_ represent the corresponding gains. We used the following equation to define the effective pressure acting on the kidneys $\text{P}_{\text{k}}$:

| $\text{P}_{\text{k}}\text{ = (MAP/}\text{S}_{\text{k}}\text{)}$ | $\text{(S33)}$ |
| --- | --- |

We obtained the normal urinary output of each kidney $\text{U}_{\text{o1}}$ by fitting cubic splines to the data given by Guyton (1980). We calculated the normal urinary output from both kidneys $\text{U}_{\text{o}}$ as follows:

| $\text{U}_{\text{o}}\text{ = }{\text{2}\text{·}\text{U}}_{\text{o1}}$ | $\text{(S34)}$ |
| --- | --- |

Before finding the effect of $\text{P}_{\text{k}}$ on the angiotensin system, we scaled it by the factor $\text{k}_{\text{ang}}$ to obtain the effective pressure acting on the angiotensin system $\text{P}_{\text{k1}}$:

| $\text{P}_{\text{k1}}\text{ =}\text{ k}_{\text{ang}}\text{·}\text{P}_{\text{k}}$ | $\text{(S35)}$ |
| --- | --- |

We calculated the concentration factor of angiotensin in the blood due to each kidney $\text{A}_{\text{c1}}$ by fitting cubic splines to the data given by Guyton (1980). We calculated the net effect of angiotensin on the circulatory components $\text{A}_{\text{c}}$ as follows:

| $\text{A}_{\text{c}}\text{ = 0.8+2}\text{·}\text{A}_{\text{c1}}$ | $\text{(S36)}$ |
| --- | --- |

We calculated the effect of angiotensin on the circulation after accounting for the delays $\text{A}_{\text{e}}$ as follows:

| $\text{A}_{\text{e}}\text{ = 1+ ∫[}\left( \text{A}_{\text{c}}\text{-}\text{A}_{\text{n}} \right)\text{/}\text{τ}\text{ang}\text{]dt}$ | $\text{(S37)}$ |
| --- | --- |

where $\text{τ}\text{ang}$ represents the time constant for the delay in development of the effect. We calculated the basic level of angiotensin activity $\text{A}_{\text{n}}$ as follows:

| $\text{A}_{\text{n}}\text{ = }\text{k}_{\text{angs}}\text{(}\text{A}_{\text{e}}\text{-1)}$ | $\text{(S38)}$ |
| --- | --- |

where $\text{k}_{\text{angs}}$ denotes the sensitivity of the angiotensin system. We calculated the effects of angiotensin on the venous unstressed blood volume A_V_, arterial resistance A_R_, and the kidney function $\text{A}_{\text{k}}$ as follows:

| $\text{A}_{\text{V}}\text{ = }\text{k}_{\text{AV}}\text{·}\text{A}_{\text{n}}$  $\text{A}_{\text{R}}\text{ = }\text{k}_{\text{AR}}\text{·}\text{A}_{\text{n}}\text{+1}$  $\text{A}_{\text{k}}\text{ = }\left[ \text{25}\text{·}\text{A}_{\text{n}}\text{+1} \right]^{\text{0.55}}$ | (S39)  (S40)  (S41) |
| --- | --- |

where the parameters k_AV_ and k_AR_ represent the gains.

We obtained the long-term $\text{A}_{\text{auto1}}$ and short-term $\text{A}_{\text{auto2}}$ autoregulation using the following integrals:

| $\text{A}_{\text{auto1}}\text{ = max(}\text{A}_{\text{min}}\text{, ∫[}\left( \text{f}_{\text{c}}\text{-}\text{A}_{\text{auto1}} \right)\text{/}\text{20000}\text{]dt})$  $\text{A}_{\text{auto2}}\text{ = max(}\text{A}_{\text{min}}\text{, ∫[}\left( \text{f}_{\text{c}}\text{-}\text{A}_{\text{auto2}} \right)\text{/}\text{10}\text{]dt})$ | (S42)  (S43) |
| --- | --- |

where the max function was used to enforce a lower limit on autoregulation. We calculated the total degree of autoregulation $\text{R}_{\text{auto}}$:

| $\text{R}_{\text{auto}}\text{ = }\left[ \text{A}_{\text{auto1}}\text{/5} \right]^{\text{5}}{\text{·}\left[ \text{A}_{\text{auto1}}\text{/5} \right]}^{\text{0.2}}$ | $\text{(S44)}$ |
| --- | --- |

We obtained the venous unstressed blood volume $\text{V}_{\text{vu}}$, arterial resistance $\text{R}_{\text{a}}$, heart strength HS, and urinary output $\text{f}_{\text{u}}$ based on the effects of sympathetic stimulation and angiotensin.

| $\text{V}_{\text{vu}}\text{ =}\text{ V}_{\text{vu0}}\text{-}\text{S}_{\text{V}}\text{-}\text{A}_{\text{V}}\text{+}\text{V}_{\text{vu0}}\text{·}\text{δ}_{\text{v}}$  $\text{HS = }\text{S}_{\text{HS}}\text{·}\text{HS}_{\text{0}}\text{·(1+}\text{δ}_{\text{h}}\text{)}$  $\text{R}_{\text{a}}\text{ = }\text{S}_{\text{R}}\text{·}\text{A}_{\text{R}}\text{·}\text{R}_{\text{auto}}\text{·(1+}\text{δ}_{\text{r}}\text{)·}\text{δ}_{\text{local}}$  $\text{f}_{\text{u}}\text{ = }\text{U}_{\text{o}}/\text{A}_{\text{k}}$ | $\text{(S45)}$  $\text{(S46)}$  $\text{(S47)}$  $\text{(S48)}$ |
| --- | --- |

where V_vu0_, R_a0_, and HS_0_ represent the initial values of the respective variables, $\text{δ}_{\text{v}}$, $\text{δ}_{\text{h}}$, and $\text{δ}_{\text{r}}$ denote the sympathetic regulation of unstressed blood volume, heart strength, and resistance, respectively, due to changes in arterial gas concentrations, and $\text{δ}_{\text{local}}$ represents the local regulation of arterial resistance due to changes in blood gas levels.

We calculated the normal heart rate $\text{HR}_{\text{n}}$ using the blood pressure of the right atrium and the regulatory effects due to sympathetic stimulation (Abram et al., 2007), as follows:

| $\text{HR =}\text{ HR}_{\text{0}}\text{·[72/(72}\text{+}\text{δ}_{\text{hs}}\text{+}\text{δ}_{\text{hv}}\text{)]+(}\text{HR}_{\text{1}}\text{·}\text{P}_{\text{ra}}\text{)+(}\text{HR}_{\text{2}}\text{·}\text{S}_{\text{s}}\text{)}$ | $\text{(S49)}$ |
| --- | --- |

where HR_1_ and HR_2_ denote are the gains, HR_0_ denotes the intercept of the equation, and $\text{δ}_{\text{hs}}$ and $\text{δ}_{\text{hv}}$ represent the regulation of heart rate due to changes in the arterial gas concentrations. Guyton’s model of cardiovascular dynamics does not explicitly compute the systolic and diastolic blood pressures (SBP and DBP). We used empirical equations derived from those of HumMod (Hester et al., 2011) to compute SBP and DBP:

| $\text{SBP = MAP+}\left[ \text{k}_{\text{bp}}\text{·(0.75CO/HR+0.25}\text{CO}_{\text{0}}\text{/}\text{HR}_{\text{0}}\text{)} \right]$ | $\text{(S50)}$ |
| --- | --- |
| $\text{DBP = MAP-}\left[ \text{k}_{\text{bp}}\text{·(0.75CO/HR+0.25}\text{CO}_{\text{0}}\text{/}\text{HR}_{\text{0}}\text{)/2} \right]$ | $\text{(S51)}$ |

where HR denotes the heart rate and $\text{k}_{\text{bp}}$ represents a coefficient.

## Respiratory system

Our model of the respiratory system was based on Cheng’s model (Cheng et al., 2010). We imposed macroscopic mass balance for oxygen and carbon dioxide in the lungs, body tissues, and the brain. Equations (S52)–(S60) describe the ventilatory dynamics by imposing macroscopic mass balances in the lungs, brain tissue, and body tissue for O_2_ and CO_2_. For the dead spaces:

| $\text{V}_{\text{d(1)}}{\dot{\text{P}}}_{\text{d}\left( \text{1} \right)\text{O2}}\text{ = }\text{MV}\text{·}\left( \text{P}_{\text{iO2}}\text{-}\text{P}_{\text{d}\left( \text{1} \right)\text{O2}} \right)$ | $\text{(S52)}$ |
| --- | --- |
| $\text{V}_{\text{d(i)}}{\dot{\text{P}}}_{\text{d}\left( \text{i} \right)\text{O2}}\text{ = }\text{MV}\text{·}\left( \text{P}_{\text{d}\left( \text{i-1} \right)\text{O2}}\text{-}\text{P}_{\text{d}\left( \text{i} \right)\text{O2}} \right)\text{, 2≤i≤5}$ | $\text{(S53)}$ |
| $\text{V}_{\text{d(1)}}{\dot{\text{P}}}_{\text{d}\left( \text{1} \right)\text{CO2}}\text{ = }\text{MV}\text{·}\left( \text{P}_{\text{iCO2}}\text{-}\text{P}_{\text{d}\left( \text{1} \right)\text{CO2}} \right)$ | $\text{(S54)}$ |
| $\text{V}_{\text{d(i)}}{\dot{\text{P}}}_{\text{d}\left( \text{i} \right)\text{CO2}}\text{ = }\text{MV}\text{·}\left( \text{P}_{\text{d}\left( \text{i-1} \right)\text{CO2}}\text{-}\text{P}_{\text{d}\left( \text{i} \right)\text{CO2}} \right)\text{, 2≤i≤5}$ | $\text{(S55)}$ |

where V_d(i)_ represents the volume of i^th^ dead space compartment, MV denotes the minute ventilation, P_d(i)O2_ and P_d(i)CO2_ represent the partial pressures of oxygen and carbon dioxide in the i^th^ compartment, respectively, ${\dot{\text{P}}}_{\text{d}\left( \text{i} \right)\text{O2}}$ and ${\dot{\text{P}}}_{\text{d}\left( \text{i} \right)\text{CO2}}$ represent the time derivatives of P_d(i)O2_ and P_d(i)CO2_, respectively, and P_iO2_ and P_iCO2_ denote the partial pressures of oxygen and carbon dioxide in the inspired air, respectively.

We calculated the change in alveolar partial pressure of oxygen ${\dot{\text{P}}}_{\text{AO2}}$ and carbon dioxide ${\dot{\text{P}}}_{\text{ACO2}}$ using the following equations:

| $\text{V}_{\text{A}}{\dot{\text{P}}}_{\text{AO2}}\text{ = }\left[ \text{MV}\text{·(1-kds)·}\left( \text{P}_{\text{d(5)O2}}\text{-}\text{P}_{\text{AO2}} \right) \right]\text{+}\left[ \text{λ}\text{·}\text{CO}\text{·}\text{(1-s)(}\text{C}_{\text{vO2}}\text{-}\text{C}_{\text{AO2}}\text{)} \right]$ | $\text{(S56)}$ |
| --- | --- |
| $\text{V}_{\text{A}}{\dot{\text{P}}}_{\text{ACO2}}\text{ = }\left[ \text{MV}\text{·(1-kds)·}\left( \text{P}_{\text{d(5)CO2}}\text{-}\text{P}_{\text{ACO2}} \right) \right]\text{+}\left[ \text{λ}\text{·}\text{CO}\text{·}\text{(1-s)(}\text{C}_{\text{vCO2}}\text{-}\text{C}_{\text{ACO2}}\text{)} \right]$ | $\text{(S57)}$ |

where V_A_ denotes the volume of the lungs, kds denotes the fraction of dead space in the lungs, s denotes the pulmonary shunt fraction, C_v_ and C_A_ denote the gas concentrations (oxygen and carbon dioxide with the subscripts O2 and CO2, respectively) in the mixed venous blood and the alveoli, respectively, and $\text{λ}$ denotes a parameter for converting blood concentration into alveolar partial pressure.

For the brain and body tissues, we computed the change in oxygen ${\dot{\text{C}}}_{\text{jO2}}$ and carbon dioxide concentrations ${\dot{\text{C}}}_{\text{jCO2}}$ using the equations below (j denotes a compartment, i.e., brain tissue or body tissue):

| $\text{V}_{\text{j}}{\dot{\text{C}}}_{\text{jO2}}\text{ = }\text{Q}_{\text{j}}\left( \text{C}_{\text{aO2}}\text{-}\text{C}_{\text{jO2}} \right)\text{-}\text{M}_{\text{jO2}}$  $\text{V}_{\text{j}}{\dot{\text{C}}}_{\text{jCO2}}\text{ = }\text{Q}_{\text{j}}\left( \text{C}_{\text{aCO2}}\text{-}\text{C}_{\text{jCO2}} \right)\text{+}\text{M}_{\text{jCO2}}$ | $\text{(S58)}$ $\text{(S59)}$ |
| --- | --- |

where V_j_ denotes the volume of compartment j and C_aO2_ and C_aCO2_ denote the concentrations of oxygen and carbon dioxide in the arterial blood, respectively. M_jO2_ and M_jCO2_ denote the metabolic rates of oxygen and carbon dioxide, respectively, and Q_j_ denotes the flow rate of blood through compartment j. We computed Q_j_ as follows:

| $\text{Q}_{\text{j}}\text{ = }\left\{ \begin{aligned} \text{0.15CO, j=brain tissue} \\ \text{0.85CO, j=body tissue} \end{aligned} \right.$ | (S60) |
| --- | --- |

We used Henry’s law to compute the partial pressures of oxygen $\text{P}_{\text{jO2}}$ and carbon dioxide $\text{P}_{\text{jCO2}}$ in the brain and body tissues:

| $\text{P}_{\text{jO2}}\text{ = }\text{C}_{\text{jO2}}\text{/}\text{α}_{\text{O2}}$ | (S61) |
| --- | --- |
| $\text{P}_{\text{jCO2}}\text{ = }\text{C}_{\text{jCO2}}\text{/}\text{α}_{\text{CO2}}$ | (S62) |

where and $\text{α}_{\text{O2}}$ and $\text{α}_{\text{CO2}}$ denote the solubility of oxygen and carbon dioxide in the body compartment j (brain tissue or body tissue), respectively.

We calculated the intermediate variables F_jO2_ and F_jCO2_ used to relate the concentration and partial pressure of blood gases, as follows:

| $\text{F}_{\text{jO2}}\text{ = }\text{P}_{\text{jO2}}\text{(1+}\text{β}_{\text{1}}\text{P}_{\text{jCO2}}\text{)/[}\text{k}_{\text{1}}\text{·(}\text{1+}\text{α}_{\text{1}}\text{P}_{\text{jCO2}}\text{)]}$ | (S63) |
| --- | --- |
| $\text{F}_{\text{jCO2}}\text{ = }\text{P}_{\text{jCO2}}\text{(1+}\text{β}_{\text{2}}\text{P}_{\text{jO2}}\text{)/}\text{[k}_{\text{2}}\text{·}\text{1+}\text{α}_{\text{2}}\text{P}_{\text{jO2}}\text{]}$ | (S64) |

where j = a or v denotes arterial or venous blood, respectively, and $\text{α}_{\text{1}}$, $\text{β}_{\text{1}}$, k_1_, $\text{α}_{\text{2}}$, $\text{β}_{\text{2}}$, k_2_, a_2_, and c_2_ denote model parameters. Furthermore, we assumed that the partial pressure of gases in the blood leaving a compartment was same as that of the partial pressure of gases in the respective compartment (equilibrium is achieved in terms of gas exchange before blood exits each compartment).

## Cardiovascular regulation due to chemoreceptors

We modeled the cardiovascular regulation due to changes in blood gas concentrations based on the model by Ursino and Magosso (Ursino and Magosso, 2000; Magosso and Ursino, 2001). We computed the chemoreceptor response to the changes in partial pressures of arterial oxygen and carbon dioxide using the following equations:

| $\text{g}_{\text{ac}}\text{= exp}\left[ \left( \text{P}_{\text{aO2}}\text{-}{\tilde{\text{P}}}_{\text{O2ac}} \right)\text{/}\text{k}_{\text{ac}} \right]$ | $\text{(S65)}$ |
| --- | --- |
| $\text{φ}_{\text{ac}}\text{ = }\text{[f}_{\text{acmax}}\text{+}\text{f}_{\text{acmin}}\text{·}\text{g}_{\text{ac}}\text{]·}\left[ \text{K}\text{·}\text{ln}\left( {\text{P}_{\text{aCO2}}}/{\text{P}_{\text{aCO20}}} \right)\text{+}\text{f}_{\text{0}} \right]\text{/[1+}\text{g}_{\text{ac}}\text{]}$ | $\text{(S66)}$ |
| $\text{K = }\left\{ \begin{aligned} \text{K}_{\text{H}}\text{, }\text{P}_{\text{aO2}}\text{≥80 } \\ \text{K}_{\text{H}}\text{-0.04}\left( \text{P}_{\text{aO2}}\text{-80} \right)\text{, 40≤}\text{P}_{\text{aO2}}\text{<80} \\ \text{K}_{\text{H}}\text{-1.6, }\text{ P}_{\text{aO2}}\text{<40 } \end{aligned} \right.$ | $\text{(S67)}$ |
| $\text{f}_{\text{ac}}\text{ }\text{=}\text{ ∫[}\text{(-}\text{f}_{\text{ac}}\text{+}\text{φ}_{\text{ac}}\text{)/}\text{τ}_{\text{ac}}\text{]dt}$ | $\text{(S68)}$ |

where$\text{g}_{\text{ac}}\text{,}$ $\text{φ}_{\text{ac}}\text{,}$ and K represent intermediate variables, f_ac_ denotes the frequency discharge in the chemoreceptor afferent fibers, f_acmin_ and f_acmax_ represent the minimum and maximum of the frequency discharge, respectively, which are also the upper and lower saturation levels of the sigmoidal function $\text{φ}_{\text{ac}}$. $\tilde{P}$_O2ac_ denotes the partial pressure of arterial oxygen at the midpoint of the sigmoidal function, P_aCO20_ denotes the initial value of the partial pressure of carbon dioxide in the arteries, and k_ac_, f_0_, K_H_, and $\tau$_ac_ denote constant parameters, with $\tau$_ac_ representing the time constant of the chemoreceptor response.

We computed the tidal volume V_T_ from the minute ventilation using the following equation:

| $\text{V}\text{T }\text{= 0.15}\left( \text{MV+1} \right)^{\text{0.65}}$ | (S69) |
| --- | --- |

We used V_T_ to compute the afferent activity of pulmonary stretch receptors $\text{φ}_{\text{ap}}$ and frequency discharge of the pulmonary receptor $\text{f}_{\text{ap}}$:

| $\text{φ}_{\text{ap}}\text{ = }\text{G}_{\text{ap}}\text{·}\text{V}_{\text{T}}$ | $\text{(S70)}$ |
| --- | --- |
| $\text{f}_{\text{ap}}\text{ }\text{=}\text{ ∫[}\text{(-}\text{f}_{\text{ap}}\text{+}\text{φ}_{\text{ap}}\text{)/}\text{τ}_{\text{ap}}\text{]dt}$ | $\text{(S71)}$ |

where G_ap_ denotes a constant gain and $\text{τ}$_ap_ denotes a time constant.

We computed the sympathetic neural activation offset terms θ_sj_, where j = h, r, or v denotes the heart strength, venous resistance, or unstressed blood volume in the veins, respectively, using the following equations:

| $\text{w}_{\text{sj}}\text{ = }\text{x}_{\text{sj}}\text{/}\left\{ \text{1+exp}\left[ \left( \text{P}_{\text{aO2}}\text{-}{\tilde{\text{P}}}_{\text{O2sj}} \right)\text{/}\text{k}_{\text{sj}} \right] \right\}$ | (S72) |
| --- | --- |
| ${\dot{\text{θ}}}_{\text{O2sj}}\text{ }\text{=}\text{ }\text{(-}\text{θ}_{\text{O2sj}}\text{+}\text{w}_{\text{sj}}\text{)/}\text{τ}_{\text{isc}}$ | (S73) |
| ${\dot{\text{θ}}}_{\text{CO2sj}}\text{ }\text{=}\text{ }\left[ \text{-}\text{θ}_{\text{CO2sj}}\text{+}\text{g}_{\text{sj}}\left( \text{P}_{\text{aCO2}}\text{-}\text{P}_{\text{aCO20}} \right) \right]\text{/}\text{τ}_{\text{cc}}$ | (S74) |
| $\text{θ}_{\text{sj}}\text{ }\text{=}\text{ }\text{θ}_{\text{sjn}}\text{-}\text{θ}_{\text{O2sj}}\text{-}\text{θ}_{\text{CO2sj}}$ | (S75) |

where w_sj_ represents an intermediate variable, x_sj_ represents the saturation of the hypoxic response, $\tilde{\text{P}}$_O2sj_ represents the partial pressure of oxygen at the midpoint of the sigmoidal function, θ_O2sj_ and θ_CO2sj_ represent the effects of O_2_ and CO_2_ on θ_sj_, respectively, $\tau$_isc_ and $\tau$_cc_ represent time constants, k_sj_ and g_sj_ represent constant parameters, and $\text{θ}$_sjn_ represents the initial value of θ_sj_.

We computed the spike frequencies of the sympathetic efferent fibers f_sj_, where j = h, r, or v denotes the heart strength, venous resistance, or unstressed blood volume in the veins, respectively, using the following equations:

| $\text{f}_{\text{sj}}\text{ = }\left\{ \begin{aligned} \text{f}_{\text{es∞}}\text{+}\left( \text{f}_{\text{es0}}\text{-}\text{f}_{\text{es∞}} \right)\text{·}\text{exp}\left[ \text{1.1×10}^{\text{-3}}\left( \text{w}_{\text{csj}}\text{f}_{\text{ac}}\text{-}\text{w}_{\text{psj}}\text{f}_{\text{ap}}\text{-}\text{θ}_{\text{sj}} \right) \right]\text{, }\text{f}_{\text{sj}}\text{<}\text{f}_{\text{esmax}}\text{ } \\ \text{f}_{\text{esmax}}\text{, }\text{f}_{\text{sj}}\text{≥}\text{f}_{\text{esmax}} \end{aligned} \right.$ | (S76) |
| --- | --- |

where w_psj_ and w_csj_ denote constant synaptic weights of pulmonary receptors and chemoreceptor afferent fibers (w_psh_ = 0), respectively, and f_es∞_, f_es0_, and f_esmax_ represent constant parameters. We computed the spike frequencies of the efferent vagal fibers $\text{f}_{\text{vf}}$ as follows:

| $\text{f}_{\text{vf}}\text{ = }\text{w}_{\text{cv}}\text{·}\text{f}_{\text{ac}}\text{-}\text{w}_{\text{pv}}\text{·}\text{f}_{\text{ap}}\text{-}\text{θ}_{\text{v}}$ | (S77) |
| --- | --- |

where w_cv_ and w_pv_ denote constant synaptic weights of chemoreceptor afferent fibers and pulmonary receptors, respectively, and $\text{θ}$_v_ denotes an offset term.

The reflex regulation affects the heart strength, unstressed blood volume in the veins, venous resistance, and heart rate. We modeled the changes in heart strength $\text{δ}_{\text{h}}$ as follows:

| $\text{σ}_{\text{h}}\text{ = }\left\{ \begin{aligned} \text{G}_{\text{h}}\text{·}\ln\left[ \text{f}_{\text{sh}}\text{-}\text{f}_{\text{esmin}}\text{+1} \right],\text{ }\text{f}_{\text{sh}}\text{≥}\text{f}_{\text{esmin}}\text{ } \\ \text{0, }\text{f}_{\text{sh}}\text{<}\text{f}_{\text{esmin }} \end{aligned} \right.$ | $\text{(S78)}$ |
| --- | --- |
| $\text{ }{\dot{\text{δ}}}_{\text{h}}\text{ = (-}\text{δ}_{\text{h}}\text{+}\text{σ}_{\text{h}}\text{)/}\text{τ}_{\text{h}}$ | $\text{(S79)}$ |

where $\sigma$_h_ denotes the output of a static characteristic, f_esmin_ denotes a threshold for sympathetic stimulation, G_h_ represents a constant gain, and $\tau$_h_ denotes a time constant.

Ursino and Magosso (2000) modeled multiple venous compartments using multiple equations. Because we modeled only a single compartment, we combined the equations from their model to represent the effect for a single compartment. We described the effect of reflex regulation on the unstressed volume and venous resistance $\text{δ}_{\text{j}}$ ($\text{j}$ = r or v denotes venous resistance or venous unstressed blood volume, respectively) using the following equations:

| $\text{σ}_{\text{j}}\text{ = }\left\{ \begin{aligned} \text{G}_{\text{j}}\text{·}\ln\left[ \text{f}_{\text{sp}}\text{-}\text{f}_{\text{esmin}}\text{+1} \right],\text{ }\text{f}_{\text{sp}}\text{≥}\text{f}_{\text{esmin}}\text{ } \\ \text{0, }\text{f}_{\text{sp}}\text{<}\text{f}_{\text{esmin}} \end{aligned} \right.$ | (S80) |
| --- | --- |
| $\dot{\text{δ}_{\text{j}}}\text{ }\text{=}\text{ }\text{(-}\text{δ}_{\text{j}}\text{+}\text{σ}_{\text{j}}\text{)/}\text{τ}_{\text{j}}$ | (S81) |

where $\text{σ}_{\text{j}}$ denotes the static output.

We used the following equations to represent the effect of reflex regulation on the heart rate:

| $\text{σ}_{\text{hs}}\text{ = }\left\{ \begin{aligned} \text{G}_{\text{hs}}\text{·}\text{ln} \left[ \text{f}_{\text{sh}}\text{-}\text{f}_{\text{esmin}}\text{+1} \right],\text{ }\text{f}_{\text{sh}}\text{≥}\text{f}_{\text{esmin}}\text{ } \\ \text{0, }\text{f}_{\text{sh}}\text{<}\text{f}_{\text{esmin}} \end{aligned} \right.$ | (S82) |
| --- | --- |
| $\dot{\text{δ}}\text{hs}\text{ }\text{=}\text{ }\text{(-}\text{δ}_{\text{hs}}\text{+}\text{σ}_{\text{hs}}\text{)/}\text{τ}_{\text{hs}}$ | (S83) |
| ${\dot{\text{δ}}}_{\text{hv}}\text{ }\text{=}\text{ }\text{(-}\text{δ}_{\text{hv}}\text{+}\text{G}_{\text{hv}}\text{·}\text{f}_{\text{v}}\text{)/}\text{τ}_{\text{hv}}$ | (S84) |

where $\text{δ}_{\text{hs}}$ and $\text{δ}_{\text{hv}}$ denote the heart rate changes due to oxygen and carbon dioxide, respectively, and HR includes the effects of changes in arterial pressure and the blood gas concentrations. We assumed that the oxygen and carbon dioxide concentrations can also change the venous resistance locally to regulate the blood flow rates as follows:

| ${\dot{\text{δ}}}_{\text{lO2}}\text{ = }\left\{ \text{g}_{\text{O2}}\left[ \text{C}_{\text{vO2}}\text{-}\text{C}_{\text{vO20}} \right]\text{-}\text{δ}_{\text{lO2}} \right\}\text{/}\text{τ}_{\text{O}_{\text{2}}}$ | $\text{(S85)}$ |
| --- | --- |
| $\text{ψ(P) = \{1-exp[(}\text{P}_{\text{aCO2}}\text{-}\text{P}_{\text{aCO20}}\text{)/}\text{k}_{\text{CO2}}\text{]\}/\{1+exp[(}\text{P}_{\text{aCO2}}\text{-}\text{P}_{\text{aCO20}}\text{)/}\text{k}_{\text{CO2}}\text{]\}}$ | $\text{(S86)}$ |
| ${\dot{\text{δ}}}_{\text{lCO2}}\text{ }\text{=}\text{ }\left[ \text{ψ(}\text{P}_{\text{aCO2}}\text{)}\text{-}\text{δ}_{\text{lCO2}} \right]\text{/}\text{τ}_{\text{CO2}}$ |  |
| $\text{δ}_{\text{local}}\text{ }\text{=}\text{ (1+}{\dot{\text{δ}}}_{\text{lCO2}}\text{)}\text{/}\text{(1+}{\dot{\text{δ}}}_{\text{lO2}}\text{)}$ | $\text{(S87)}$ |

where $\text{δ}_{\text{lO2}}$ and $\text{δ}_{\text{lCO2}}$ denote the contribution of oxygen and carbon dioxide to the local regulation of resistance, respectively, $\text{g}_{\text{O2}}$ and $\text{k}_{\text{CO2}}$ denote constant parameters, $\text{C}_{\text{vO20}}$ denotes the initial concentration of oxygen in the veins, and $\text{τ}$_O2_ and $\text{τ}$_CO2_ denote time constants.

We assumed that the minute ventilation was regulated as follows:

| $\text{G}_{\text{ventc}}\text{ = }\left\{ \begin{aligned} \text{1.8, }\text{P}_{\text{aCO2}}\text{≥}\text{P}_{\text{aCO2n}}\text{ } \\ \text{0.12, }\text{P}_{\text{aCO2}}\text{≤}\text{P}_{\text{aCO2n}} \end{aligned} \right.$ | $\text{(S88)}$ |
| --- | --- |
| $\dot{\text{δ}}\text{ventp}\text{ }\text{=}\text{ [0.06}\text{(}\text{f}_{\text{ac}}\text{-}\text{f}_{\text{acn}}\text{)-}\text{δ}_{\text{ventp}}\text{]/}\text{τ}_{\text{ventp}}$ | $\text{(S89)}$ |
| $\dot{\text{δ}}\text{ventc}\text{ }\text{=}\text{ [}\text{G}_{\text{ventc}}\text{(}\text{f}_{\text{ac}}\text{-}\text{f}_{\text{acn}}\text{)-}\text{δ}_{\text{ventc}}\text{]/}\text{τ}_{\text{ventc}}$ | $\text{(S90)}$ |
| $\text{MV}\text{ }\text{= }\text{MV}_{\text{0}}\text{+}\text{(δ}_{\text{ventp}}\text{+}\text{δ}_{\text{ventc}}\text{+1)}\text{·}\text{MV}_{\text{0}}\text{/7}$ | $\text{(S91)}$ |

where $\text{G}_{\text{ventc}}$ represents the gain of the central mechanism, $\text{δ}_{\text{ventp}}$ denotes the regulation of ventilation due to peripheral feedback, $\text{δ}_{\text{ventc}}$ represents the regulation of ventilation due to the central mechanism, $\text{τ}$ denotes time constants, and $\text{P}_{\text{aCO2n}}$ and $\text{f}_{\text{acn}}$ represent the nominal values of partial pressure of carbon dioxide in the arteries and the frequency discharge in the chemoreceptor afferent fibers, respectively. $\text{MV}_{\text{0}}$ denotes the baseline value of minute ventilation. If the ventilation was forced (using a ventilator), we set $\text{MV}$ equal to the corresponding value.

# Model parameters

Table S1. Complete set of parameters used in the cardio-respiratory model

1. Cardiovascular model

| **P**  **#** | **Parameter name** | **Description** | **Value** | **Units** |
| --- | --- | --- | --- | --- |
| 1 | V_V0_ | Initial blood volume in the veins | 3.257 | L |
| 2 | V_Vu0_ | Initial unstressed blood volume in the veins | 2.950 | L |
| 3 | C_v_ | Venous compliance | 8.25$\text{×}$10^−2^ | L/mmHg |
| 4 | R_v0_ | Initial venous resistance | 0.740 | mmHg$\text{·}$min/L |
| 5 | V_ra0_ | Initial blood volume in the right atrium | 0.100 | L |
| 6 | V_rau_ | Unstressed blood volume in the right atrium | 0.100 | L |
| 7 | C_ra_ | Right atrial compliance | 0.005 | L/mmHg |
| 8 | V_a0_ | Initial blood volume in the arteries | 0.850 | L |
| 9 | V_au_ | Unstressed blood volume in the arteries | 0.495 | L |
| 10 | C_a_ | Arterial compliance | 3.60$\text{×}$10^−3^ | L/mmHg |
| 11 | R_a0_ | Initial arterial resistance | 19.340 | mmHg$\text{·}$min/L |
| 12 | HS_0_ | Initial heart strength | 1.000 | - |
| 13 | k_SV_ | Gain, sympathetic effects on V_V0_ | 0.250 | L |
| 14 | k_SHS_ | Gain, sympathetic effects on HS | 1.000 | - |
| 15 | k_SR_ | Gain of the sympathetic effects on R_a0_ | 1.000 | - |
| 16 | k_Sk_ | Gain of the sympathetic effects on kidney function | 1.000 | - |
| 17 | k_AV_ | Gain of the angiotensin effects on V_V0_ | 0.200 | L |
| 18 | k_AR_ | Gain of the angiotensin effects on R_a0_ | 1.000 | - |
| 19 | HR_0_ | Intercept in the equation for HR computation | 32.000 | beats/min |
| 20 | HR_1_ | Gains in the equation for HR computation | 2.000 | beats/  (min$\text{·}$mmHg) |
| 21 | HR_2_ | Gains in the equation for HR computation | 40.000 | beats/min |
| 22 | $\text{k}\text{cp}$† | Factor used in the calculation of capillary pressure | 2.800 | mmHg$\text{·}$min/L |
| 23 | $\text{k}_{\text{ab}}$† | Factor used to scale the pressure acting on the baroreceptor | 0.650 | - |
| 24 | $\text{k}_{\text{sym}}$† | Sensitivity of the sympathetic stimulation | 1.500 | - |
| 25 | $\text{k}_{\text{ang}}$† | Factor used to scale the pressure acting on the angiotensin system | 0.450 | - |
| 26 | $\text{k}_{\text{angs}}$† | Sensitivity of the angiotensin system | 1.200 | - |
| 27 | $\text{A}_{\text{min}}$† | Lower limit of autoregulation | 2.190 | L/min |
| 28 | $\text{σ}^{\text{salt}}$† | Reflection coefficient of salt | 0.013 | - |
| 29 | $\text{σ}^{\text{alb}}$ | Reflection coefficient of albumin | 0.950 | - |
| 30 | $\text{L}_{\text{Pl-EC}}$ | Hydraulic conductivity of endothelial cells | 8.49$\text{×}$10^−10^ | L/(min $\text{·}$mmHg$\text{·}$cm^2^) |
| 31 | $\text{L}_{\text{Pl-ISS}}$† | Hydraulic conductivity of pores | 1.35$\text{×}$10^−6^ | L/(min $\text{·}$mmHg$\text{·}$cm^2^) |
| 32 | $\text{L}_{\text{Pl-RBC}}$ | Hydraulic conductivity of red blood cells | 7.20$\text{×}$10^−10^ | L/(min $\text{·}$mmHg$\text{·}$cm^2^) |
| 33 | $\text{L}_{\text{ISS-Tcell}}$ | Hydraulic conductivity of tissue cells | 1.30$\text{×}$10^−9^ | L/(min $\text{·}$mmHg$\text{·}$cm^2^) |
| 34 | $\text{S}_{\text{Pl-EC}}$ | Surface area of the endothelial cell layer | 2.94$\text{×}$10^6^ | cm^2^ |
| 35 | $\text{S}_{\text{Pl-ISS}}$ | Surface area of pores | 2.94$\text{×}$10^4^ | cm^2^ |
| 36 | $\text{S}_{\text{Pl-RBC}}$ | Surface area of red blood cells | 1.35$\text{×}$10^2^ | μm^2^/cell |
| 37 | $\text{S}_{\text{ISS-Tcell}}$ | Surface area of tissue cells | 8.82$\text{×}$10^6^ | cm^2^ |
| 38 | $\text{J}_{\text{L0}}^{\text{w}}$ | Lymphatic flow at nominal conditions | 1.60$\text{×}$10^-3^ | L/min |
| 39 | $\text{P}\text{S}_{\text{Pl-ISS}}^{\text{salt}}$ | Permeability surface area product of salt | 2.500 | L/min |
| 40 | $\text{P}\text{S}_{\text{Pl-ISS}}^{\text{alb}}$† | Permeability surface area product of albumin | 2.50$\text{×}$10^-3^ | L/min |
| 41 | $\text{V}_{\text{refill}}^{\text{max}}$† | Upper bound on transcapillary refill | 0.938 | L |
| 42 | k_bp_ | Coefficient for systolic and diastolic pressure | 11.600 | mmHg$\text{·}$beats/L |
| 43 | $\text{τ}\text{sym}$† | Time constant, stimulated sympathetic activity | 0.261 | min |
| 44 | $\text{τ}\text{ang}$† | Time constant, angiotensin response | 6.443 | min |

†Parameter value estimated during model calibration.
Remaining parameter values were obtained from the literature (Guyton, 1980; Mazzoni et al., 1988; Jin et al., 2023).

1. Respiratory model

| **P**  **#** | **Parameter name** | **Description** | **Value** | **Units** |
| --- | --- | --- | --- | --- |
| 45 | V_d(i)_, i = 1,2,…,5 | Volume of i^th^ dead space compartment | 0.030 | L |
| 46 | V_A_ | Volume of the lungs | 6.300 | L |
| 47 | s | Shunt fraction | 0.038 | - |
| 48 | V_b_ | Volume of the brain tissue | 1.255 | L |
| 49 | V_d_ | Volume of the body tissue | 42.000 | L |
| 50 | M_bO2_ | O_2_ consumption rate in the brain tissue | 0.038 | L/min |
| 51 | M_dO2_ | O_2_ consumption rate in the body tissue | 0.260 | L/min |
| 52 | M_bCO2_ | CO_2_ production rate in the brain tissue | 0.038 | L/min |
| 53 | M_dCO2_ | CO_2_ production rate in the body tissue | 0.170 | L/min |
| 54 | $\text{α}_{\text{1}}$ | Parameters in the equation for O_2_ and CO_2_ pressures and concentrations | 0.040 | 1/mmHg |
| 55 | $\text{β}_{\text{1}}$ |  | 0.008 | 1/mmHg |
| 56 | k_1_ |  | 14.000 | mmHg |
| 57 | $\text{α}_{\text{2}}$ |  | 0.056 | 1/mmHg |
| 58 | $\text{β}_{\text{2}}$ |  | 0.032 | 1/mmHg |
| 59 | k_2_ |  | 1.94$\text{×}$10^2^ | mmHg |
| 60 | kds | Fraction of dead space in the lung | 0.350 | - |
| 61 | $\text{λ}$ | Coefficient that converts blood concentration into alveolar partial pressure | 8.63$\text{×}$10^2^ | mmHg |
| 62 | $\text{α}_{\text{O2}}$ | O_2_ solubility in the brain and body tissues | 3.00$\text{×}$10^-5^ | 1/mmHg |
| 63 | $\text{α}_{\text{CO2}}$ | CO_2_ solubility in the brain and body tissues | 7.00$\text{×}$10^-4^ | 1/mmHg |
| 64 | $\text{a}_{\text{1}}$ | Coefficients in the equation for O_2_ and CO_2_ pressures and concentrations | 0.384 | - |
| 65 | $\text{a}_{\text{2}}$ |  | 1.800 | - |
| 66 | $\text{c}_{\text{1}}$ |  | 9.000 | mmol/L |
| 67 | $\text{c}_{\text{2}}$ |  | 86.110 | mmol/L |

All parameter values were obtained from the literature (Cheng et al., 2010; Briesenick et al., 2023).

1. Control due to chemoreceptors

| **P**  **#** | **Parameter name** | **Description** | **Value** | **Units** |
| --- | --- | --- | --- | --- |
| 68 | f_acmin_ | Upper saturation level of frequency discharge in the chemoreceptor afferent | 50.000 | spikes/min |
| 69 | f_acmax_ | Lower saturation level of frequency discharge in the chemoreceptor afferent | 7.38$\text{×}$10^2^ | spikes/min |
| 70 | $\tilde{\text{P}}$_O2ac_ | Arterial O_2_ pressure at the central point of the afferent chemoreceptor | 45.000 | mmHg |
| 71 | k_ac_ | Parameters in the afferent chemoreceptor response | 29.270 | mmHg |
| 72 | f_0_ |  | 1.400 | - |
| 73 | K_H_ |  | 3.000 | - |
| 74 | G_ap_ | Gain of the pulmonary receptor response | 1.40$\text{×}$10^3^ | spikes/min/L |
| 75 | x_sh_ | Parameters in the offset terms of the sympathetic neural activation equation | 3.18$\text{×}$10^3^ | 1/min |
| 76 | $\tilde{\text{P}}$_O2sh_ |  | 45.000 | mmHg |
| 77 | k_sh_ |  | 6.000 | mmHg |
| 78 | g_sh_ |  | 60.000 | 1/(mmHg$\text{·}$min) |
| 79 | x_sr_, x_sv_ |  | 3.60$\text{×}$10^2^ | 1/min |
| 80 | $\tilde{\text{P}}$_O2sr_, $\tilde{\text{P}}$_O2sv_ | Parameters in the offset terms of the sympathetic neural activation equation | 30.000 | mmHg |
| 81 | k_sr_, k_sv_ |  | 2.000 | mmHg |
| 82 | g_sr_ |  | 90.000 | 1/(mmHg$\text{·}$min) |
| 83 | g_sv_ |  | 0.000 | 1/(mmHg$\text{·}$min) |
| 84 | $\text{θ}$_shn_ | Initial offset terms of the sympathetic neural activation equation | 2.16$\text{×}$10^2^ | 1/min |
| 85 | $\text{θ}$_srn_, $\text{θ}$_svn_ | Initial offset term of the sympathetic neural activation equation | 7.99$\text{×}$10^2^ | 1/min |
| 86 | f_es0_ | Parameters for the frequencies of spikes in the sympathetic efferent fibers | 9.67$\text{×}$10^2^ | spikes/min |
| 87 | f_esmax_ |  | 3.60$\text{×}$10^3^ | spikes/min |
| 88 | f_es∞_ |  | 1.26$\text{×}$10^2^ | spikes/min |
| 89 | w_csp_, w_csv_ |  | 5.000 | - |
| 90 | w_csh_ |  | 1.000 | - |
| 91 | w_psp_, w_psv_ |  | -0.340 | - |
| 92 | w_cv_ |  | 0.200 | - |
| 93 | w_pv_ |  | 0.103 | - |
| 94 | $\text{θ}$_v_ |  | -40.800 | spikes/min |
| 95 | f_esmin_ | Threshold for sympathetic stimulation | 1.60$\text{×}$10^2^ | spikes/min |
| 96 | G_h_ | Gain factor for change of heart strength | 3.80$\text{×}$10^3^ | - |
| 97 | G_r_ | Gain factor for change of venous resistance | 1.04$\text{×}$10^2^ | mmHg$\text{·}$min$\text{/}$L |
| 98 | G_v_† | Gain factor for change of unstressed blood volume in the veins | -0.159 | L |
| 99 | G_hs_ | Gain factor for change of heart rate | -2.20$\text{×}$10^-3^ | beats/min |
| 100 | G_hv_ |  | 1.50$\text{×}$10^-3^ | beats/min |
| 101 | $\text{k}_{\text{CO2}}$ | Parameters for local blood control | 1.43$\text{×}$10^3^ | mmHg |
| 102 | $\text{g}_{\text{O2}}$ |  | 3.000 | mL/mL |
| 103 | $\text{τ}$_ac_ | Time constants for the chemoreceptor response | 0.033 | min |
| 104 | $\text{τ}$_ap_ |  | 0.033 | min |
| 105 | $\text{τ}$_isc_ | Time constants for the offset terms | 0.500 | min |
| 106 | $\text{τ}$_cc_ |  | 0.330 | min |
| 107 | $\text{τ}$_h_ | Time constant for change of heart strength | 0.133 | min |
| 108 | $\text{τ}$_r_ | Time constant for change of venous resistance | 0.100 | min |
| 109 | $\text{τ}$_v_ | Time constant for change of unstressed blood volume in the veins | 0.330 | min |
| 110 | $\text{τ}$_hs_ | Time constants for change of heart rate | 0.033 | min |
| 111 | $\text{τ}$_hv_ |  | 0.025 | min |
| 112 | $\text{C}_{\text{vO20}}$ | Nominal value, oxygen concentration in veins | 0.134 | mL/mL |
| 113 | $\text{τ}$_O2_ | Time constants for local blood flow control | 0.167 | min |
| 114 | $\text{τ}$_CO2_ |  | 0.333 | min |
| 115 | $\text{τ}_{\text{ventp}}$ | Time constants for regulation of ventilation | 0.217 | min |
| 116 | $\text{τ}_{\text{ventc}}$ |  | 3.000 | min |
| 117 | $\text{P}_{\text{aCO2n}}$ | Nominal value, partial pressure of arterial carbon dioxide | 30.340 | mmHg |
| 118 | $\text{f}_{\text{acn}}$ | Nominal value, $\text{f}_{\text{acn}}$ | 81.397 | spikes/min |

†Parameter value estimated during model calibration.
Remaining parameter values were obtained from the literature (Ursino and Magosso, 2000; Magosso and Ursino, 2001).

# Experimental procedure of *Study 4*

*Study 4* was conducted by the U.S. Army Institute of Surgical Research (USAISR) in Fort Sam Houston, Texas, at a facility certified by the Association for the Assessment and Accreditation of Laboratory Animal Care International. The study was approved by the USAISR Institutional Animal Care and Use Committee and was performed in compliance with the Animal Welfare Act and in accordance with the Guide for the Care and Use of Laboratory Animals. In this study, 108 Yorkshire-cross female pigs with an average weight of 40.3 kg (standard deviation = 2.4 kg) (Midwest Swine Research, Gibbon, MN) were used to investigate the effects of hemorrhage (controlled and uncontrolled) and fluid resuscitation with three different fluid types administered at three different rates (i.e., standard, slow, and bolus). The animals were divided into nine treatment groups with 12 animals per group. As previously described in detail by Soller et al. (2014) and Sondeen et al. (2011), the following procedures were performed for each of the treatment groups.

**3.1 Surgical instrumentation**

The pigs were fasted 12 to 18 h before surgery, with water available *ad libitum*. Before surgery, the pigs received intramuscular injections of glycopyrrolate (Robinul, 0.01 mg/kg; Baxter Healthcare, Deerfield, IL) and tiletamine-zolazepam (Telazol, 8 mg/kg; Wyeth, Fort Dodge, IA) for saliva secretion control and sedation, respectively. Anesthesia was induced via a facemask with approximately 5% isoflurane (Forane; Baxter Healthcare) in 100% oxygen and was maintained during surgical instrumentation with 1% to 3% isoflurane in 30% oxygen in air using a ventilator and monitor (Apollo; Draeger Medical, Telford, PA). Animals were placed in the supine position, and electrocardiogram electrodes were attached to monitor their heart rate (HR). A pressure transducer-tipped catheter (Mikro-Tip; Millar Instruments, Inc., Houston, TX) was placed in the carotid artery for blood pressure monitoring, a Swan Ganz catheter was advanced into the pulmonary artery via a jugular vein for measuring cardiac output, and a catheter was placed in a jugular vein for blood sampling. Additional catheters were placed in the left femoral artery and vein for arterial hemorrhage and intravenous infusion of the resuscitation fluids. A laparotomy was performed to access the spleen. Suction tubes with perforated tips were placed in the peritoneal cavity to collect blood from the injured spleen. Ventilation was adjusted to maintain an end-tidal PCO_2_ of approximately 40 mmHg. Core temperature was maintained between 37°C and 39°C.

**3.2 Experimental procedure**

After instrumentation was completed and mean arterial pressure (MAP) stabilized, a baseline period was maintained for 10 min during which hemodynamic measurements, including blood pressure, cardiac output, HR, hemoglobin concentration, and oxygen delivered, were recorded. All the data from the analog signals were collected on a data acquisition instrumentation rack/biomedical data recorder and physiological data recorder program (Dynamic Research Evaluation Workstation-DREW, USAISR, San Antonio, TX). After blood was drawn for baseline measurements, a controlled hemorrhage was performed by removing 24 mL/kg blood at a rate of 100 mL/min, which was typically completed in ~10 min. After the controlled hemorrhage, a splenic injury was made by transecting the spleen along the long axis, offset 1 cm from the midline to avoid large vessels, and the uncontrolled hemorrhage volume was measured continuously by suctioning the shed blood into canisters placed on a balance. A blood sample was collected at the end of the initial 15-min blood loss from the splenic injury. Next, resuscitation fluid [normal saline (NS), fresh frozen plasma (FFP), and fresh whole blood (FWB)] was provided at three different rates: *1*) standard infusion, where NS was infused at 1.5 ml/(kg$\text{·}$min) and both FFP and FWB were infused at 1 ml/(kg$\text{·}$min); *2*) slow continuous infusion, where NS was infused at 0.32 ml/(kg$\text{·}$min), FFP was infused at 0.24 ml/(kg$\text{·}$min), and FWB was infused at 0.07 ml/(kg$\text{·}$min); and *3*) bolus infusion, where NS was infused at 4.5 ml/(kg$\text{·}$min) and both FFP and FWB were infused at 2.8 ml/(kg$\text{·}$min). For all three infusion rates, the total volume of NS provided was 45 ml/kg and the total volume of both FFP and FWB provided was 15 ml/kg. Animals were monitored for 5 hours after the splenic injury or until death, and no additional fluid was given. In addition to the baseline blood sample, arterial and venous samples were drawn for analysis at 15, 30, 60, 90, 120, 240, and 300 min after injury or at death if the animal did not survive.

**3.3 Power analysis**

A power analysis (PASS; NCSS, Inc.) was performed to estimate the sample size needed to detect a 50% decrease in bleeding rate with a standard deviation of 40% and resulted in a sample size of 12 animals per group. To detect differences among the means versus the alternative of equal means, the total sample size of 108 animals achieved 83.6% power using an F test with a 0.05 significance level.

**3.4 Data analysis**

At the BHSAI, we received the USAISR data for all 108 animals from the nine treatment groups. For our analysis, we only used the data from animals that survived for at least 60 min or longer after the start of the uncontrolled hemorrhage. For each of the five measured hemodynamic variables, MAP, cardiac output, HR, hemoglobin concentration, and delivered oxygen, in each treatment group (n = ~12), we calculated the means and standard errors of the mean at time points representing the end of the baseline period, the beginning and end of the controlled and uncontrolled hemorrhage periods (these time points varied between individual animals), as well as at 15, 30, and 60 min after the start of the uncontrolled hemorrhage. We simulated each of the nine experimental treatment scenarios and compared our model predictions of the five variables with the corresponding experimental data. Finally, we calculated the root mean square errors between the model predictions and the experimental mean values for these variables, which we reported in Table 2.

# Supplementary figures


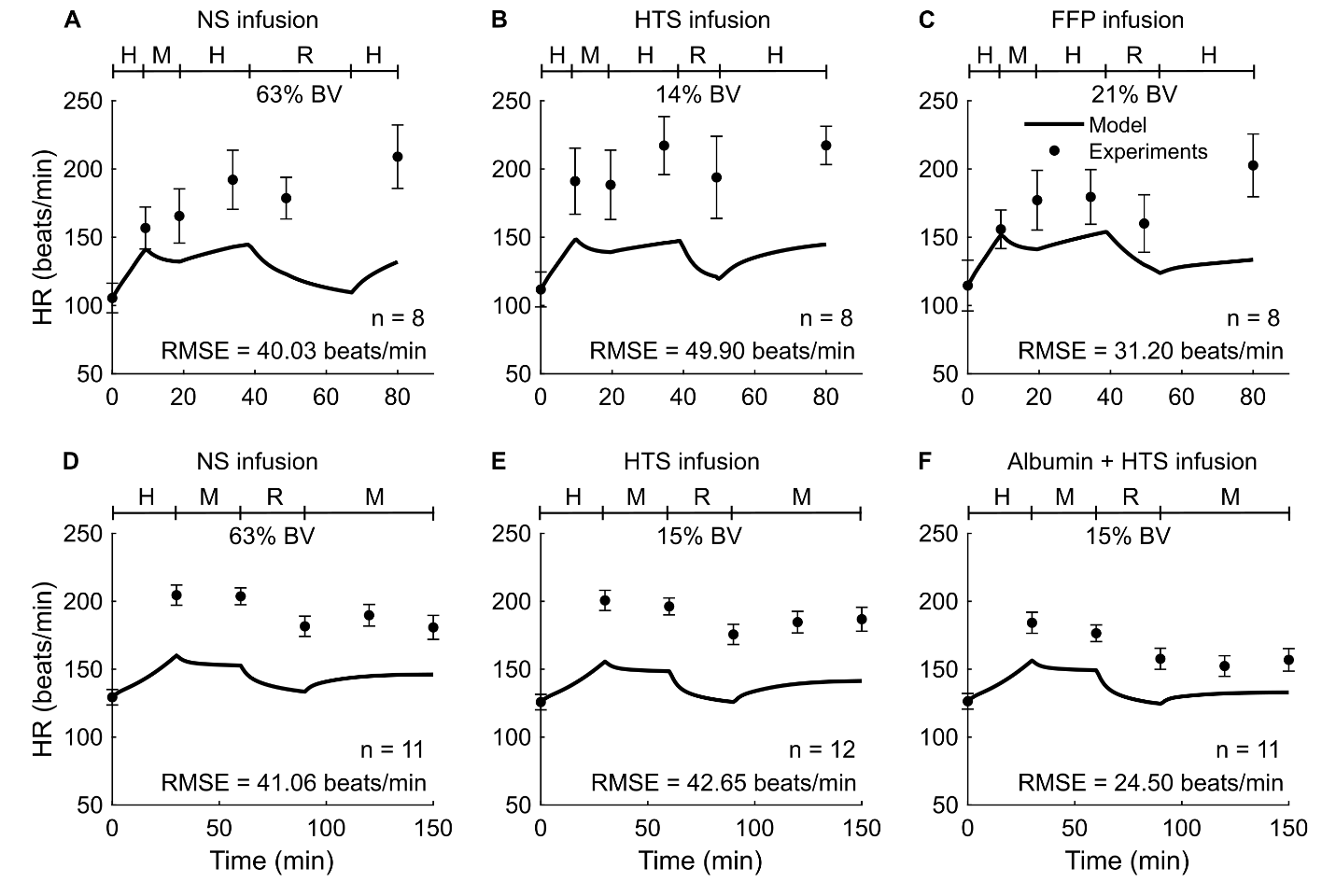


Figure S1. Assessment of model fit (calibration) in *Study 1* (**A-C**) and model prediction (validation) in *Study 3* (**D-F**) for heart rate (HR). The left panels show the results for infusion of normal saline (NS), the middle panels for 3% hypertonic saline (HTS), and the right panels for either fresh frozen plasma (FFP) or 5% albumin plus 3% hypertonic saline (Albumin + HTS). The timeline at the top of each subplot illustrates the hemorrhagic shock protocol, wherein H represents the hemorrhage period, M denotes the monitoring period, and R represents the resuscitation period. The error bars denote two standard errors of the mean. BV: blood volume; RMSE: root mean square error.


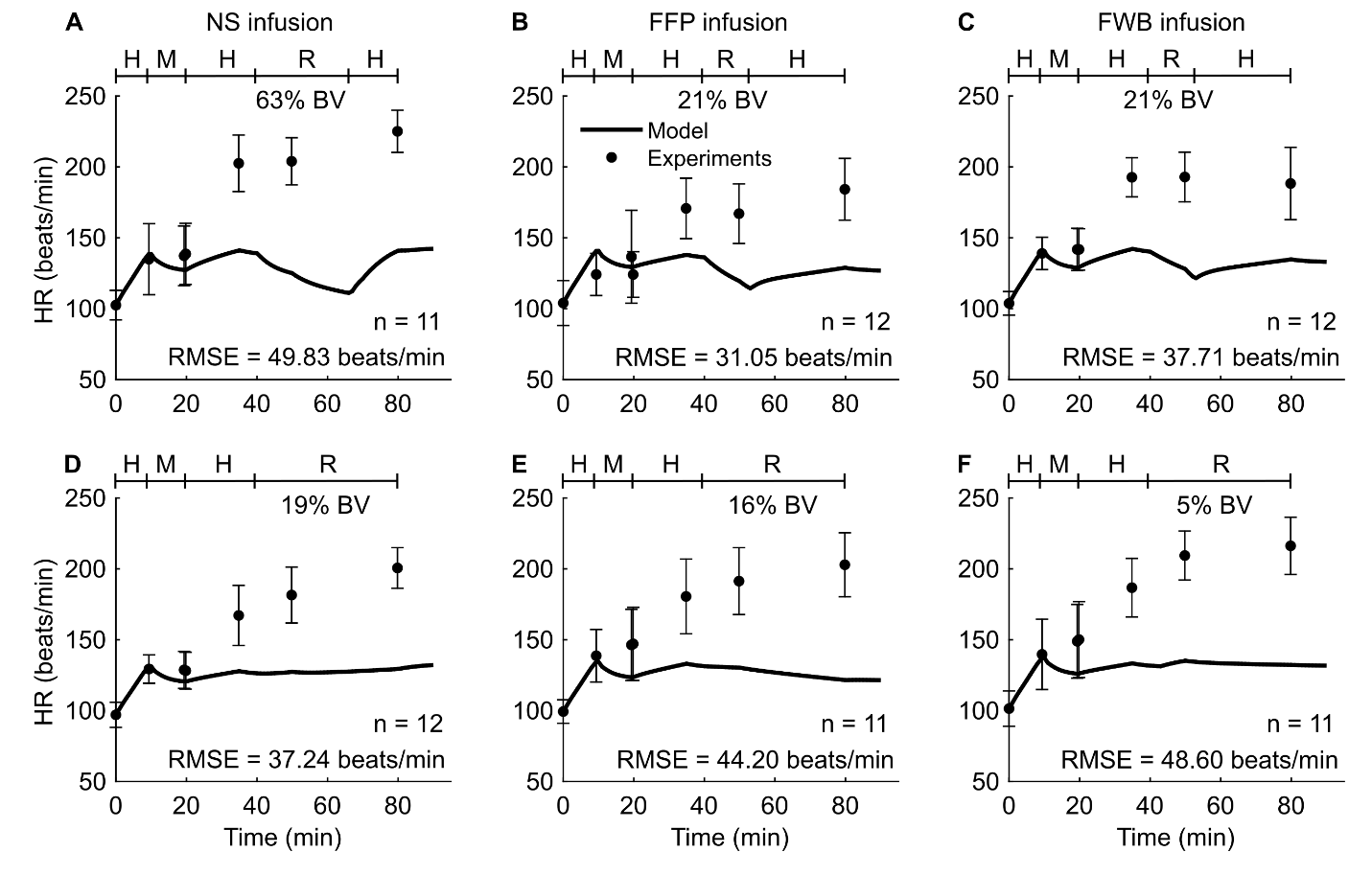


Figure S2. Assessment of model prediction (validation) of heart rate (HR) for *Study 4*. (**A-C**) HR values for standard infusion and (**D-F**) HR values for slow infusion. The left panels show the results for infusion of normal saline (NS), the middle panels for fresh frozen plasma (FFP), and the right panels for fresh whole blood (FWB). The timeline at the top of each subplot illustrates the hemorrhagic shock protocol, wherein H represents the hemorrhage period, M denotes the monitoring period, and R represents the resuscitation period. The error bars denote two standard errors of the mean. BV: blood volume; RMSE: root mean square error.

# References

Abram, S.R., Hodnett, B.L., Summers, R.L., Coleman, T.G., and Hester, R.L. (2007). Quantitative circulatory physiology: an integrative mathematical model of human physiology for medical education. *Advances in Physiology Education* 31(2), 202-210. doi: 10.1152/advan.00114.2006.

Briesenick, L., Schaade, A., Bergholz, A., Hoppe, P., Kouz, K., Krause, L., et al. (2023). Energy expenditure under general anesthesia: an observational study using indirect calorimetry in patients having noncardiac surgery. *Anesthesia & Analgesia* 137(1), 169-175. doi: 10.1213/ane.0000000000006343.

Cheng, L., Ivanova, O., Fan, H.-H., and Khoo, M.C. (2010). An integrative model of respiratory and cardiovascular control in sleep-disordered breathing. *Respiratory Physiology & Neurobiology* 174(1-2), 4-28. doi: 10.1016/j.resp.2010.06.001.

Guyton, A.C. (1980). *Arterial Pressure and Hypertension.* Philadelphia, PA: Saunders.

Hester, R.L., Brown, A.J., Husband, L., Iliescu, R., Pruett, D., Summers, R., et al. (2011). HumMod: a modeling environment for the simulation of integrative human physiology. *Frontiers in Physiology* 2, 12. doi: 10.3389/fphys.2011.00012.

Jin, X., Laxminarayan, S., Nagaraja, S., Wallqvist, A., and Reifman, J. (2023). Development and validation of a mathematical model to simulate human cardiovascular and respiratory responses to battlefield trauma. *International Journal for Numerical Methods in Biomedical Engineering* 39(1), e3662. doi: 10.1002/cnm.3662.

Magosso, E., and Ursino, M. (2001). A mathematical model of CO_2_ effect on cardiovascular regulation. *American Journal of Physiology-Heart and Circulatory Physiology* 281(5), H2036-H2052. doi: 10.1152/ajpheart.2001.281.5.H2036.

Mazzoni, M.C., Borgstrom, P., Arfors, K.-E., and Intaglietta, M. (1988). Dynamic fluid redistribution in hyperosmotic resuscitation of hypovolemic hemorrhage. *American Journal of Physiology-Heart and Circulatory Physiology* 255(3), H629-H637. doi: 10.1152/ajpheart.1988.255.3.H629.

Soller, B., Smith, C., Zou, F., Ellerby, G.E., Prince, M.D., and Sondeen, J.L. (2014). Investigation of noninvasive muscle pH and oxygen saturation during uncontrolled hemorrhage and resuscitation in swine. *Shock* 42(1), 44-51. doi: 10.1097/SHK.0000000000000174.

Sondeen, J.L., Prince, M.D., Kheirabadi, B.S., Wade, C.E., Polykratis, I.A., de Guzman, R., et al. (2011). Initial resuscitation with plasma and other blood components reduced bleeding compared to hetastarch in anesthetized swine with uncontrolled splenic hemorrhage. *Transfusion* 51(4), 779-792. doi: 10.1111/j.1537-2995.2010.02928.x.

Ursino, M., and Magosso, E. (2000). Acute cardiovascular response to isocapnic hypoxia. I. A mathematical model. *American Journal of Physiology-Heart and Circulatory Physiology* 279(1), H149-H165. doi: 10.1152/ajpheart.2000.279.1.H149.
